# Genome-Wide Identification and Expression Analysis of the WRKY Gene Family in *Eucalyptus grandis* Under Drought Stress During Arbuscular Mycorrhizal Symbiosis

**DOI:** 10.3390/microorganisms14081626

**Published:** 2026-07-25

**Authors:** Yanjing Yu, Yuxin Zhong, Siyuan Li, Yuanli Tu, Xi Liu, Sijia Wang

**Affiliations:** Guangxi Key Laboratory of Forest Ecology and Conservation, Guangxi Colleges and Universities Key Laboratory for Cultivation and Utilization of Subtropical Forest Plantation, School of Forestry, Guangxi University, Nanning 530004, China

**Keywords:** *Arbuscular mycorrhizal* fungi, drought stress, WRKY gene family, RNA-seq analysis

## Abstract

*Eucalyptus* is an important timber species characterized by strong adaptability and rapid growth. However, adverse environmental conditions such as drought stress severely impact its growth and productivity. Arbuscular mycorrhizal (AM) fungi form beneficial symbiotic relationships with *Eucalyptus* root systems and significantly enhance plant stress tolerance. In this study, we identified 111 WRKY genes in *Eucalyptus grandis* and systematically characterized their physicochemical properties, phylogenetic relationships, gene structures, conserved motifs, synteny, and cis-acting elements. Notably, AM fungal symbiosis significantly enhanced the biomass, plant height, and root length of *E. grandis* seedlings under drought stress. Through integrated RNA-seq and qRT-PCR analyses, we identified 12 *EgWRKY* genes that responded to drought stress during AM fungal symbiosis, with their expression levels significantly elevated in AM-inoculated roots under drought conditions. These findings provide novel insights into the regulatory roles of *EgWRKY* genes in AM-mediated drought tolerance and establish a foundation for understanding the molecular mechanisms underlying WRKY-mediated stress responses in *E. grandis*.

## 1. Introduction

The WRKY gene family is one of the largest families of transcription factors (TFs) identified in plants. WRKY proteins possess a conserved WRKYGQK domain and a zinc finger structure [1]. Based on the number of WRKY domains and the type of zinc finger motifs, they can be classified into three major groups and multiple subgroups [2]. These proteins have been identified in various plant species, including *Arabidopsis thaliana* [3], *Oryza sativa* [4], *Glycine max* [5], *Populus trichocarpa* [6], *Cucumis sativus* [7], and *Zea mays* [8]. Members of this family play crucial regulatory roles in plant growth and development, biomass formation, secondary metabolite synthesis, and responses to biotic and abiotic stresses. When plants face abiotic stresses (such as drought, salt stress, temperature fluctuations, and nutrient deficiency), WRKY transcription factors act as signaling nodes to regulate the balance between plant growth and stress responses [9,10].

WRKY transcription factors play a significant role in plant drought response. Under drought stress, plants can enhance their drought tolerance by accumulating oligosaccharides through sucrose metabolism. When *A. thaliana* is subjected to drought stress, the expression of *AtWRKY53*, which binds to the Qua-Quine Starch (QQS) promoter sequence, is rapidly induced. This leads to a decrease in hydrogen peroxide levels and a significant enhancement of sugar metabolism pathways, thereby regulating stomatal opening and ultimately influencing drought tolerance [11]. WRKY transcription factors also regulate plant stress tolerance through abscisic acid (ABA) and reactive oxygen species (ROS) related signaling pathways. *GhWRKY1bD* promotes stomatal closure by enhancing ABA accumulation, regulates the activity of ROS-metabolizing enzymes to reduce oxidative damage, and simultaneously upregulates *P5CS* expression to promote proline synthesis, thereby synergistically enhancing the drought tolerance of *Gossypium hirsutum* through multiple pathways [12]. Furthermore, WRKY proteins can directly regulate the expression of drought-responsive genes. *SbWRKY30* (*Sorghum bicolor*) acts as a positive regulator of drought resistance by binding to the W-box element in the promoter of the drought-resistant gene *SbRD19*, thereby inducing *SbRD19* expression and enhancing the plant’s drought tolerance [13]. *Arabidopsis* Group IId WRKYs (*AtWRKY11*, *AtWRKY17*) suppress the expression of stress-responsive genes by forming WRKY-OBE complexes, thereby maintaining normal growth. Under drought stress, their expression is repressed, activating stress-responsive genes to enhance drought tolerance [14]. Group III WRKYs (*WRKY46*, *WRKY54*, *WRKY70*) regulate drought responses via the brassinolide (BR) signaling pathway, promoting the expression of growth-related genes and suppressing drought-resistant genes [15]. WRKYs are also involved in drought adaptation during seed germination (*AtWRKY57*, *GmWRKY16*), root development (*OsWRKY71*), and the reproductive stage (*OsWRKY11*) [16,17,18,19,20]. Some WRKYs (*AtWRKY30*) can even simultaneously enhance drought tolerance and biomass, providing new insights for crop breeding [21].

The mechanisms underlying plant drought tolerance are not only reflected in the plant’s own regulatory processes but are also closely related to the role of rhizosphere microorganisms [22]. As a key component of the rhizosphere, these microorganisms significantly influence the interactions between plants and the soil environment, playing a positive role in promoting nutrient uptake, regulating immune responses, and enhancing plant productivity and tolerance to environmental stress [23,24]. AM fungi are a type of beneficial symbiotic fungi associated with plant roots, primarily consisting of spores, hyphae, vesicles, and arbuscules [25]. When AM fungi establish symbiotic associations with plant roots, they develop an extensive hyphal network connecting the rhizosphere with the root epidermis. This network effectively enhances forest tree productivity by improving growth and development, water status, and nutrient accumulation, and to some extent mitigates the adverse effects of drought stress on forest trees [26]. AM fungal symbiosis can also enhance tree drought resistance by influencing molecular pathways. Studies on improving drought resistance in *Solanum lycopersicum* and *Z. mays* have found that AM fungal symbiosis can enhance plant drought resistance and improve water relations by regulating 14-3-3 genes in the ABA signaling pathway [27,28]. Numerous studies have confirmed the establishment of mycorrhiza in *E. grandis*, and this symbiotic relationship plays a crucial role in promoting plant growth and enhancing productivity [29,30]. Compared to the non-inoculated control, mycorrhizal-inoculated *E. grandis* seedlings exhibited significantly enhanced growth under organic nitrogen and phosphorus fertilization. AM fungal symbiosis improves nitrogen use efficiency, thereby increasing nitrogen content and enhancing photosynthesis in seedlings, which in turn promotes seedling growth [31]. Concurrently, AM fungal symbiosis can also maintain ROS homeostasis in *E. grandis* seedlings under drought stress by enhancing antioxidant capacity, thereby reducing cellular damage and alleviating the adverse effects of drought stress [32].

*E. grandis* is a plant belonging to the genus *Eucalyptus* within the family Myrtaceae, widely utilized in various sectors including structural timber, engineered wood products, biomass energy, and pulp and paper production. With ongoing climate change, extreme weather events such as heatwaves, heavy rainfall, storms, and drought stress are becoming increasingly frequent worldwide [33,34]. Affected by drought stress, *E. grandis* plantations have exhibited a continued decline in growth rate in recent years [35,36,37]. Although genome-wide identification of the EgWRKY family has been previously conducted, and the expression of *EgWRKY* genes under AM fungal colonization coupled with infection by the bacterial pathogen (*Ralstonia solanacearum*) has been investigated [38,39], the expression patterns of these transcription factors under AM symbiosis during drought stress remain largely unexplored.

In this study, 111 *EgWRKY* genes were identified from the *E. grandis* genome, and their physicochemical properties, phylogeny, gene structure, conserved motifs, cis-acting elements, chromosomal localization, gene duplication, and synteny were comprehensively analyzed using bioinformatics. Furthermore, RNA-seq and qRT-PCR were used to analyze the expression patterns of *EgWRKY* genes under drought stress in both AM-inoculated and non-inoculated plants. This study provides valuable insights into the identification of key genes involved in the mechanisms by which *EgWRKY* transcription factors mediate drought tolerance of *E. grandis* under AM fungal symbiosis.

## 2. Materials and Methods

### 2.1. Genome-Wide Identification and Physicochemical Analysis of WRKY Genes in E. grandis

The whole-genome sequence, coding sequences (CDS), protein sequences, and annotation files for *E. grandis* were obtained from the NCBI database (ASM1654582v1, https://www.ncbi.nlm.nih.gov/datasets/genome/GCF_016545825.1/ (accessed on 7 November 2025)) [40]. Two methods were used to identify the *E. grandis* WRKY gene family: 1. Protein sequences from the AtWRKY, ZmWRKY, and GmWRKY were downloaded from the NCBI database. Using these protein sequences as templates, the BLASTP tool in the TBtools-II v2.454 was employed to preliminarily screen for EgWRKY protein sequences (score value  ≥  100, e-value  ≤  1 × 10^−10^) [41]. 2. The Hidden Markov Model (HMM) file for the WRKY domain (PF03106) was downloaded from the Pfam 38.2 protein family database (http://pfam.xfam.org/ (accessed on 7 November 2025)) [8]. We then used TBtools-II v2.454 to identify WRKY proteins in the *E. grandis* genome database. Finally, we combined the BLASTP and HMM results and selected the 113 genes in the intersection for candidate gene identification. Subsequently, the online tools InterPro (https://www.ebi.ac.uk/interpro/search/sequence/ (accessed on 7 November 2025)), SMART (http://smart.embl.de/ (accessed on 7 November 2025)), and NCBI CDD (https://www.ncbi.nlm.nih.gov/cdd/ (accessed on 7 November 2025)) were used to verify whether the EgWRKY proteins possessed conserved WRKY domains [42]. After excluding two candidate proteins lacking conserved WRKY domains, a total of 111 EgWRKY proteins were identified. The physicochemical properties of the EgWRKY proteins, including molecular weight (MW), theoretical isoelectric point (pI), and hydrophilicity, were calculated using the ExPASy server (https://www.ExPASy.org/(accessed on 8 November 2025)) with the default parameters [43]. The subcellular localization of EgWRKY family members was predicted using the WoLF PSORT server (https://wolfpsort.hgc.jp/ (accessed on 8 November 2025)) [44].

### 2.2. Multiple Sequence Alignment and Phylogenetic Analysis of WRKY Proteins in E. grandis

Multiple sequence alignments of EgWRKY and AtWRKY protein sequences were performed using the ClustalW tool in MEGA 11.0, and the results were exported as FASTA files. Subsequently, sequence alignment diagrams were generated using ESPript 3.2 (https://espript.ibcp.fr/ESPript/cgi-bin/ESPript.cgi (accessed on 17 November 2025)). A phylogenetic tree was constructed using MEGA 11.0 (Neighbor-Joining method) with 1000 bootstrap replicates (All other parameters were set to their default values) [45]. Visualization and annotation of the generated tree file were performed using iTOL (https://itol.embl.de/ (accessed on 17 November 2025)).

### 2.3. Analysis of the Gene Structures, Conserved Motifs, and Cis-Acting Elements of WRKY Genes in E. grandis

Based on the genome annotation data, the gene structures (intron–exon organization) of the *EgWRKY* genes were analyzed using TBtools-II v2.454. The MEME Suite (https://meme-suite.org/meme/doc/meme.html (accessed on 18 November 2025)) was used to identify conserved motifs in the EgWRKY proteins. The maximum number of conserved motifs was set to 10, and all other parameters were left at their default values [46]. Subsequently, the gene structures and conserved motifs were visualized using TBtools-II v2.454 (All other parameters were set to their default values). Cis-acting elements in the promoter region of the *EgWRKY* genes were predicted using the PlantCARE website (upstream 2000 base pairs) (http://bioinformatics.psb.ugent.be/webtools/plantcare/html/ (accessed on 18 November 2025)) [47].

### 2.4. Chromosomal Mapping and Collinearity Analysis of WRKY Genes in E. grandis

Based on the gene annotation files of the *E. grandis* genome, a chromosomal map of the EgWRKY gene family was constructed using the TBtools-II v2.454. The MCScanX tool in TBtools-II v2.454 was used to analyze tandem and segmental duplication events in the *EgWRKY* genes with default parameters. Based on the EgWRKY protein sequences and CDS, the Ka, Ks, and Ka/Ks values for the *EgWRKY* genes were calculated using TBtools-II v2.454. The MCScanX tool was used to analyze the collinearity between *E. grandis* and five other plant species (*O. sativa*, *Z. mays*, *A. thaliana*, *P. trichocarpa*, *Malus domestica*), and the results were subsequently visualized using TBtools-II v2.454.

### 2.5. Plant Materials, Drought Stress Treatments, and Tissue Collection

In this experiment, seeds of *E. grandis* were obtained from the Institute of Tropical Forestry, Chinese Academy of Forestry Sciences. The *E. grandis* seeds were sterilized with 3% sodium hypochlorite for 20 min, then rinsed three times with sterile distilled water. The seeds were germinated for 7 days in sterilized quartz sand at 25 °C. The AM fungal species used in this study was *Rhizophagus irregularis* (DAOM-197198). The propagation host plant was *Trifolium repens*. After germination for two weeks, the clover was inoculated with the fungal inoculum, and the substrate was quartz sand. Long-Ashton (LA) nutrient solution was applied twice per week [48]. Three months after inoculation, the shoots were removed, and the surface sand and coarse roots adjacent to the shoots were discarded. The remaining roots were chopped and mixed with the underlying sand to form a mixture containing fungal spores, hyphae, root fragments, and quartz sand, which was used for subsequent plant inoculation.

After seed germination, seedlings with uniform growth were selected and transplanted into small plastic pots (8 cm × 8 cm × 8 cm), where they were inoculated with AM fungi at a rate of 50 mL of AM fungal inoculum per pot. The pots were then transferred to a growth chamber with a 16 h light/8 h dark cycle, at 26 °C during the light period and 20 °C during the dark period, until the *E. grandis* seedlings were well-established. Drought treatment was initiated 42 days after inoculation, during which Long-Ashton medium (LA) was applied twice weekly. Two moisture gradients were established: WW (Well water), with 75% field water capacity (FWC); and DS (drought stress), with 25% FWC [49]. Under each moisture condition, two sub-treatments were established: arbuscular mycorrhizal (AM) and non-mycorrhizal (NM), resulting in a total of 4 treatments with 3 replicates per treatment. After 14 days of drought treatment, root samples from the seedlings were collected, immediately frozen in liquid nitrogen, and stored at −80 °C.

### 2.6. Measurement of Biomass and Mycorrhizal Colonization in E. grandis Seedlings

At harvest, the shoot fresh weight and root fresh weight of seedlings subjected to arbuscular mycorrhizal (AM) and non-mycorrhizal (NM) treatments were measured using an electronic balance, and plant height and root length were measured with a tape measure. After gently blotting the roots dry to remove surface moisture, they were placed in a 10% sodium hydroxide (KOH) solution and incubated in a water bath at 90 °C for 4–6 h (which could be extended if necessary) until the pigment deposits in the *E. grandis* root system were released and the color of the KOH solution lightened; the KOH solution was replaced with fresh solution every hour. Subsequently, the roots were immersed in 2% hydrochloric acid (HCl) for 10 min to neutralize the KOH, then rinsed three times with sterile water. Afterward, the roots were stained with a 0.05% trypan blue solution at 37 °C in the dark for 30 min, and the root tissue was then transferred to a buffer solution. The buffer solution consisted of Hank’s balanced salt solution (HBSS) and glycerol (1:1, *v*/*v*). Finally, MYCOCALC program was used to quantify the mycorrhizal colonization by Arbuscular mycorrhizae [50].

### 2.7. Transcriptomic Analysis of the WRKY Gene Family in E. grandis

Transcriptome sequencing, based on the Illumina platform, involves the analysis of transcriptome profiles in specific tissues or cells at a given time point. It provides a foundation for studying gene function and regulatory mechanisms and plays an important role in understanding organismal growth and development [51]. In this study, samples from different treatment groups (each group comprising three biological replicates) were subjected to transcriptome sequencing performed by Novogene. RNA integrity and quantity were assessed using the Agilent 2100 Bioanalyzer (Agilent Technologies, Santa Clara, CA, USA). mRNA was enriched using Oligo (dT) magnetic beads or rRNA depletion strategies, fragmented using divalent cations in NEB Fragmentation Buffer, and sequencing libraries were constructed using either the NEB standard or strand-specific protocol [52]. After quantification by Qubit 2.0 Fluorometer and insert size verification by Agilent 2100 Bioanalyzer, the effective library concentration was determined by qRT-PCR (>1.5 nM). After passing quality control, different libraries were pooled according to effective concentration and target data volume requirements, and sequenced on an Illumina Nova X-plus platform to generate 150 bp paired-end reads. The sequencing principle was based on sequencing by synthesis (SBS). Sequencing fragments were converted to sequence data (reads) in FASTQ format by CASAVA base calling. The number of raw reads per sample ranged from 40.15 million to 50.29 million (mean: 46.22 million; total: 554.65 million across 12 samples). Adapter-containing reads, reads containing N, and low-quality reads (where bases with Phred quality ≤ 5 accounted for more than 50% of the read length) were removed. After filtering, sequencing error rate checking, and GC content distribution analysis, clean reads were obtained. Clean reads were mapped to the reference genome (ASM1654582v1) using HISAT2 [53]. Reads with mapping quality below 10, non-properly paired reads, and reads mapped to multiple regions of the genome were filtered out. Gene expression levels were quantified using featureCounts in the subread package [54], and normalized to FPKM values to account for sequencing depth and gene length differences [55]. Differential expression analysis was performed using DESeq2, with screening criteria of |log2 (FoldChange)| ≥ 1 and adjusted *p*-value (padj) ≤ 0.05 (Benjamini–Hochberg) [56]. RNA-seq expression values were transformed as log2 (FPKM + 1) and analyzed using two-way ANOVA and Tukey’s test (*p* < 0.05). Heatmaps were generated using TBtools-II v2.454.

### 2.8. Analysis of WRKY Gene Expression in E. grandis Under Drought Conditions in the Presence of AM Fungal Symbiosis Using qRT-PCR

Total RNA was extracted from seedling samples subjected to different treatments using a plant RNA extraction kit (OMEGA-R6827-01, Norcross, GA, USA), and the RNA concentration and quality were determined using a spectrophotometer. Using the extracted total RNA as a template, first-strand cDNA was synthesized with the HiScript I/II First-Strand cDNA Synthesis Kit (R233, Vazyme, Nanjing, China) and stored at −20 °C. Gene-specific primers were designed using NCBI Primer-BLAST (https://www.ncbi.nlm.nih.gov/tools/primer-blast/index.cgi?LINK_LOC=BlastHome (accessed on 25 January 2026)); the primer sequences shown in Table S6. *EgUBI3* were used as the reference gene [32,57,58,59]. qRT-PCR reactions were performed on a Roche 480 II real-time quantitative PCR instrument, with the following reaction program: 30 s pre-denaturation at 95 °C, 10 s denaturation at 95 °C, 30 s annealing at 60 °C, for 40 cycles. Melting curve analysis was performed after the reaction: denaturation at 95 °C for 5 s, followed by a temperature ramp from 65 °C to 95 °C at 0.5 °C increments with a 10 s hold at each step. Three technical replicates were performed for each sample. Relative gene expression levels were calculated using the 2^−ΔΔCt^ formula [60].

### 2.9. Statistical Analyses

All data were statistically analyzed using IBM SPSS Statistics 27. Data are presented as mean ± standard error (SE) of three biological replicates. Statistical analyses were performed using one-way or two-way analysis of variance (ANOVA), as appropriate, followed by Tukey’s test. Different letters above the bars indicate significant differences at *p* < 0.05.

## 3. Results

### 3.1. Identification and Analysis of the Physicochemical Properties of the WRKY Gene Family in E. grandis

Using protein sequences from the AtWRKY, ZmWRKY, and GmWRKY gene families, along with the hidden Markov model (HMM) file for the WRKY domain (PF03106), 113 members of the EgWRKY gene family were initially identified in the *E. grandis* genome through BLASTP and HMM methods. Subsequently, candidate sequences that were incomplete or lacking the conserved WRKY domain were filtered out (Figure S1). Ultimately, 111 *EgWRKY* genes were identified in the *E. grandis* genome. These genes were named according to their chromosomal locations and were designated *EgWRKY1*–*EgWRKY111* (Table S1).

Significant variation was observed among these 111 EgWRKY proteins, with protein lengths ranging from 139 aa (*EgWRKY5*) to 1831 aa (*EgWRKY61*), and an average protein sequence length of 456 aa. The molecular weights of the proteins ranged from 16,144.02 Da (*EgWRKY5*) to 204,607.1 Da (*EgWRKY61*), with an average of 44,500.69 Da. The isoelectric points ranged from 4.75 (*EgWRKY79*) to 11.00 (*EgWRKY73*). Forty-seven EgWRKY proteins had a pI > 7, while 64 had a pI < 7, indicating that this gene family is enriched in acidic amino acids. The average GRAVY score indicated that EgWRKY proteins are hydrophilic (Table S1).

Analysis of subcellular localization predictions using WoLF PSORT revealed that most EgWRKY proteins are localized to the nucleus (101/111, 90.99%), consistent with their putative roles as transcription factors. Four proteins were predicted to be localized to chloroplasts (*EgWRKY37*, *EgWRKY61*, *EgWRKY69*, *EgWRKY70*); one to the endoplasmic reticulum (*EgWRKY52*); one to the cytoplasm (*EgWRKY43*); two to peroxisomes (*EgWRKY11*, *EgWRKY40*); and two to the Golgi apparatus (*EgWRKY62*, *EgWRKY67*).

### 3.2. Phylogenetic Analysis and Multiple Sequence Alignment of EgWRKY Proteins

**Figure 1:**
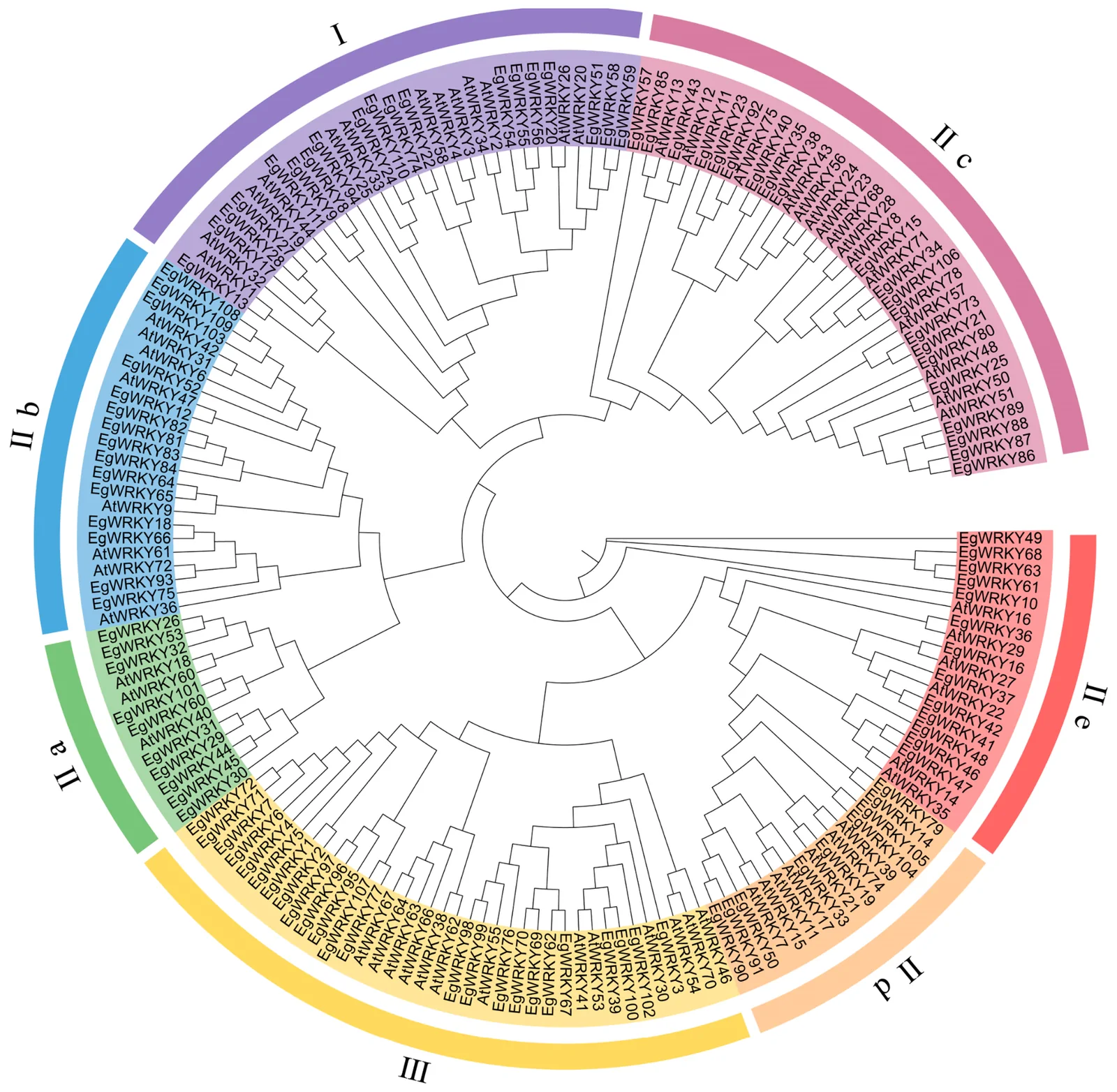
Phylogenetic analysis of WRKY proteins from *E. grandis* and *A. thaliana*. A phylogenetic tree was constructed using the neighbor-joining method with MEGA 11.0, with 1000 bootstrap replicates. Seven major subgroups (I, IIa, IIb, IIc, IId, IIe, and III) are represented by differently colored regions.

Phylogenetic analysis classified the 111 EgWRKY proteins into seven subgroups (I, IIa, IIb, IIc, IId, IIe, and III), consistent with the established *Arabidopsis* WRKY classification system (Figure 1). The EgWRKY proteins were divided into three groups (I–III), with Group II further subdivided into five subgroups: IIa, IIb, IIc, IId, and IIe. Among these, Group III contained the largest number of EgWRKY members (24), followed by Subgroup IIc (21), Group I (19), Subgroup IIb (15), Subgroup IIe (13), Subgroup IIa (10), and Subgroup IId (9). The phylogenetic tree showed that multiple EgWRKY proteins cluster closely with AtWRKY proteins, indicating that these proteins were highly homologous.

**Figure 2:**
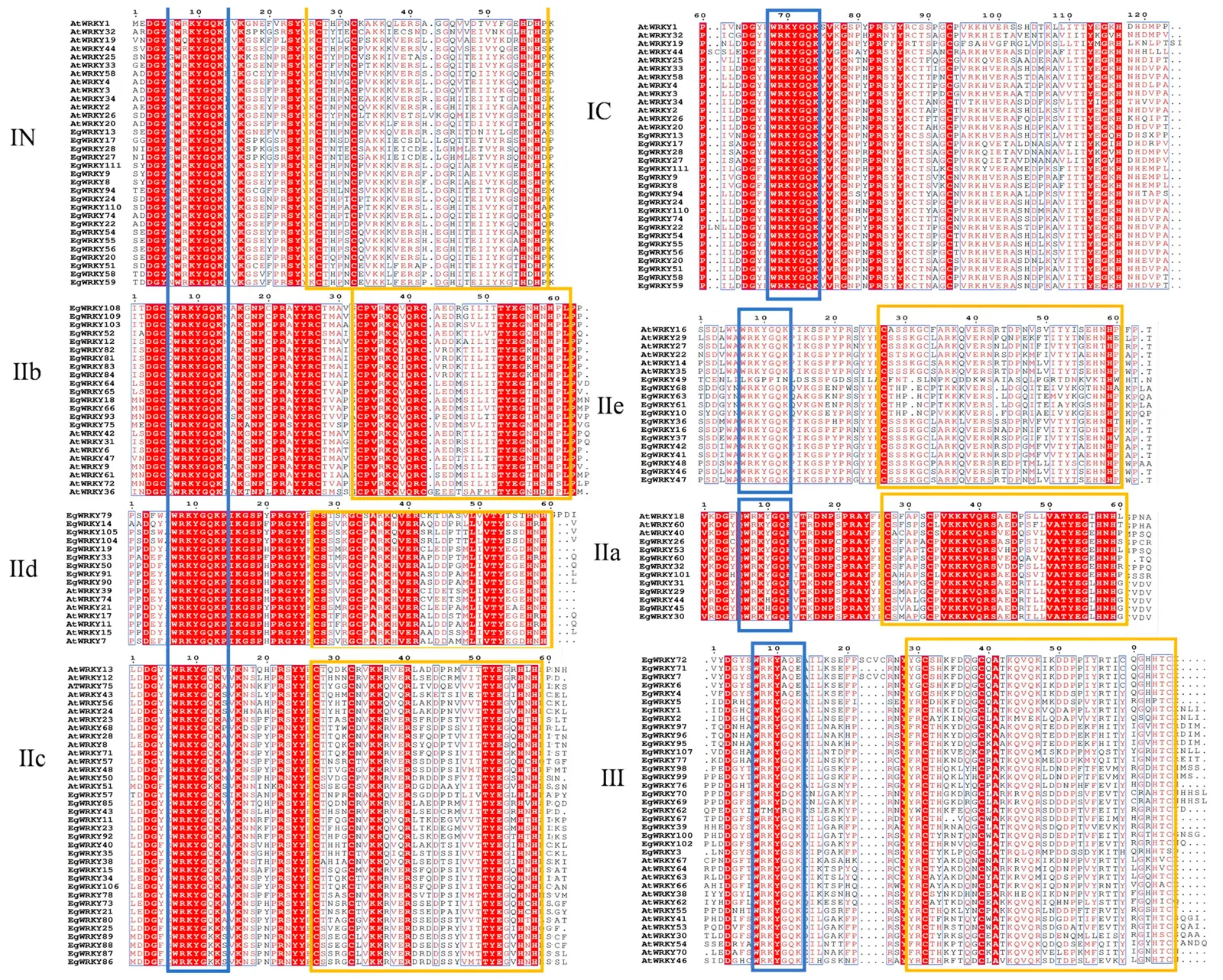
Multiple sequence alignment of WRKY domains from seven subgroups of *E. grandis* and *A. thaliana*. “N” and “C” indicate the N-terminal and C-terminal WRKY domains, respectively. The blue boxes indicate the WRKY domain, and the yellow boxes represent the zinc finger structure.

Sequence alignment of the EgWRKY domains revealed the conserved WRKYGQK heptapeptide and C_2_H_2_/C_2_HC zinc finger structures across all seven subgroups, with subgroup-specific variations observed in the zinc finger spacing (Figure 2). All members of Group I possessed two conserved WRKY domains and a C_2_H_2_ zinc finger structure (C-X_4-5_-C-X_22-23_-H-X_1_-H). Group II members all possessed one conserved WRKY domain and one C_2_H_2_-type zinc finger structure (C-X_4-5_-C-X_22-23_-H-X_1_-H). Based on phylogenetic relationships and structure, they were further divided into five subgroups: IIa, IIb, IIc, IId, and IIe. Group III members all possessed one conserved WRKY domain and one C_2_HC-type zinc finger structure (C-X_7_-C-X_23_-H-X_1_-C) [3]. A total of 93 EgWRKY proteins were found to possess the highly conserved heptapeptide sequence WRKYGQK. In addition, multiple heptapeptide variants were identified. In Subgroup IIa, the variant WKKHGQK was observed in *EgWRKY44* and *EgWRKY45*. In Subgroup IIc, the variant WRKYGKK was observed in *EgWRKY25*, *EgWRKY86*, *EgWRKY87*, *EgWRKY88*, and *EgWRKY89*. In Subgroup IIe, the variant WRKYGQR was observed in *EgWRKY68*. Furthermore, *EgWRKY49* exhibited a domain deletion. In Group III, the following variants were identified: WRKYAQE (*EgWRKY4*, *EgWRKY6*, *EgWRKY7*, *EgWRKY71*, and *EgWRKY72*); WRKYDQK (*EgWRKY69* and *EgWRKY70*); WTMRQR (*EgWRKY62*); and WRKYGSK (*EgWRKY3*).

### 3.3. Analysis of the Gene Structure, Conserved Motifs, and Cis-Acting Elements of EgWRKY Genes

**Figure 3:**
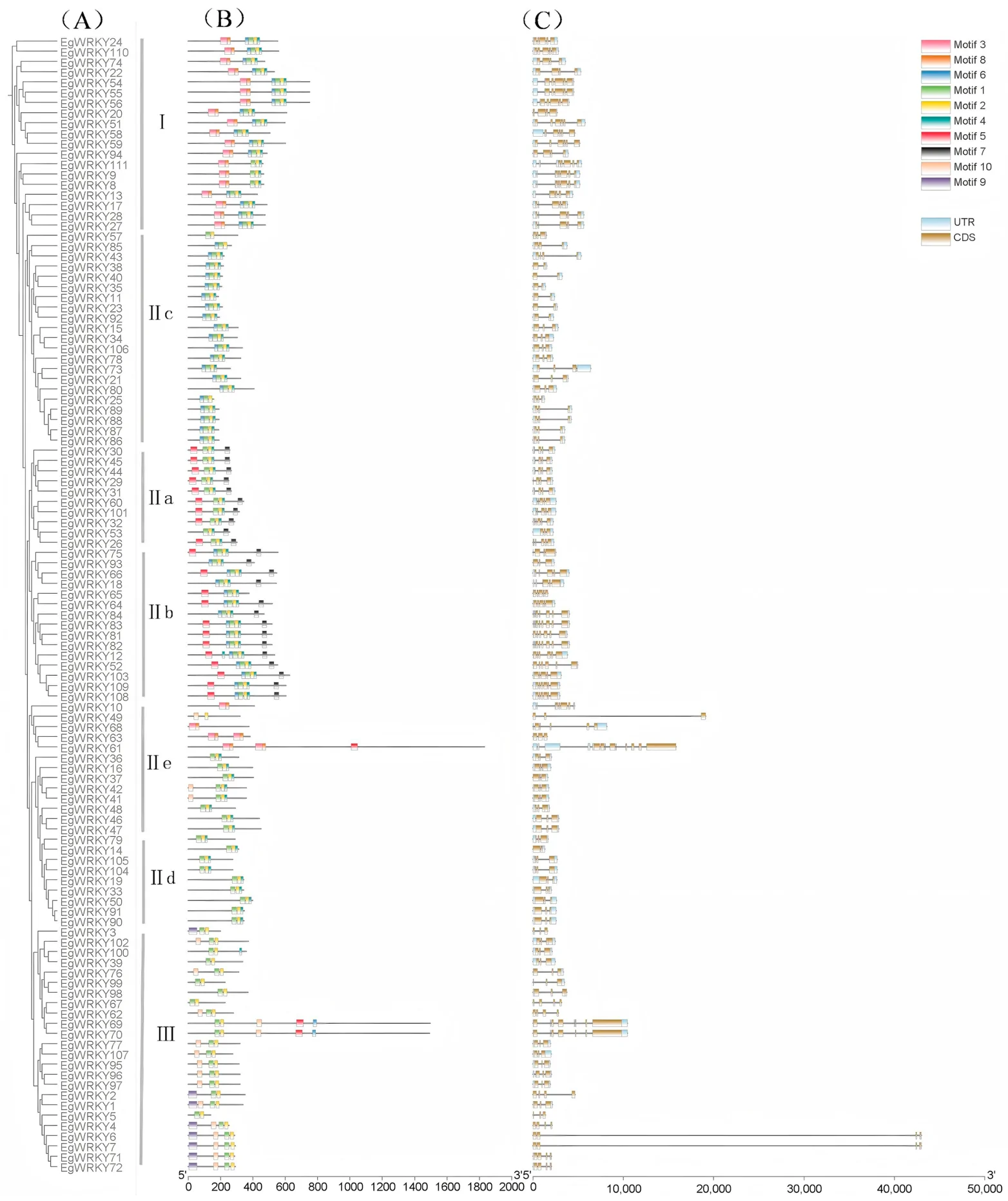
Gene structures and conserved motifs of the *EgWRKY* genes. (**A**) Phylogenetic analysis of EgWRKY proteins. (**B**) Conservative motifs (designated 1–10) in EgWRKY proteins are shown as 10 colored boxes; the horizontal black line indicates the relative protein length. (**C**) Gene structures of the *EgWRKY* genes. Light blue rectangles, brown rectangles, and black lines represent untranslated regions (UTRs), coding sequences (CDSs), and introns, respectively.

Based on the genome annotation data, the exon–intron structures of the *EgWRKY* genes were analyzed. A comparison of the number and positions of exons and introns revealed that all 111 *EgWRKY* genes contained varying numbers of exons (Figure 3C). With the exception of *EgWRKY2*, *EgWRKY3*, *EgWRKY4*, *EgWRKY5*, *EgWRKY49*, *EgWRKY62*, *EgWRKY67*, the remaining 104 *EgWRKY* genes contained untranslated regions (UTRs). Among the 111 *EgWRKY* genes, 43 (38.73%) contained 2 introns; 22 (19.81%) contained 3; 15 (13.51%) contained 4; 9 (8.11%) contained 1; and 22 (19.81%) contained 5 to 12 introns.

To further understand the evolutionary process of the EgWRKY gene family, we analyzed the conserved motifs of EgWRKY proteins in conjunction with the EgWRKY protein phylogeny (Figure 3B, Figure S2, Table S2). First, the MEME tool was used to identify 10 conserved motifs (designated motifs 1 to 10) in EgWRKY proteins. The identified motifs were visualized using TBtools-II v2.454, which showed that all 111 EgWRKY proteins contained conserved motifs. Most EgWRKY family members within the same group or subgroup shared similar conserved motifs, but some differences exist between different subgroups. Furthermore, members of the same group or subgroup possessed specific conserved motifs. For example, motif 8 was detected only in Group I, motif 7 only in Subgroups IIa and IIb, and motif 9 only in Group III. All EgWRKY proteins contained either motif 1 or motif 3, representing the conserved WRKY domain, although several members possessed variant WRKY heptapeptide sequences.

**Figure 4:**
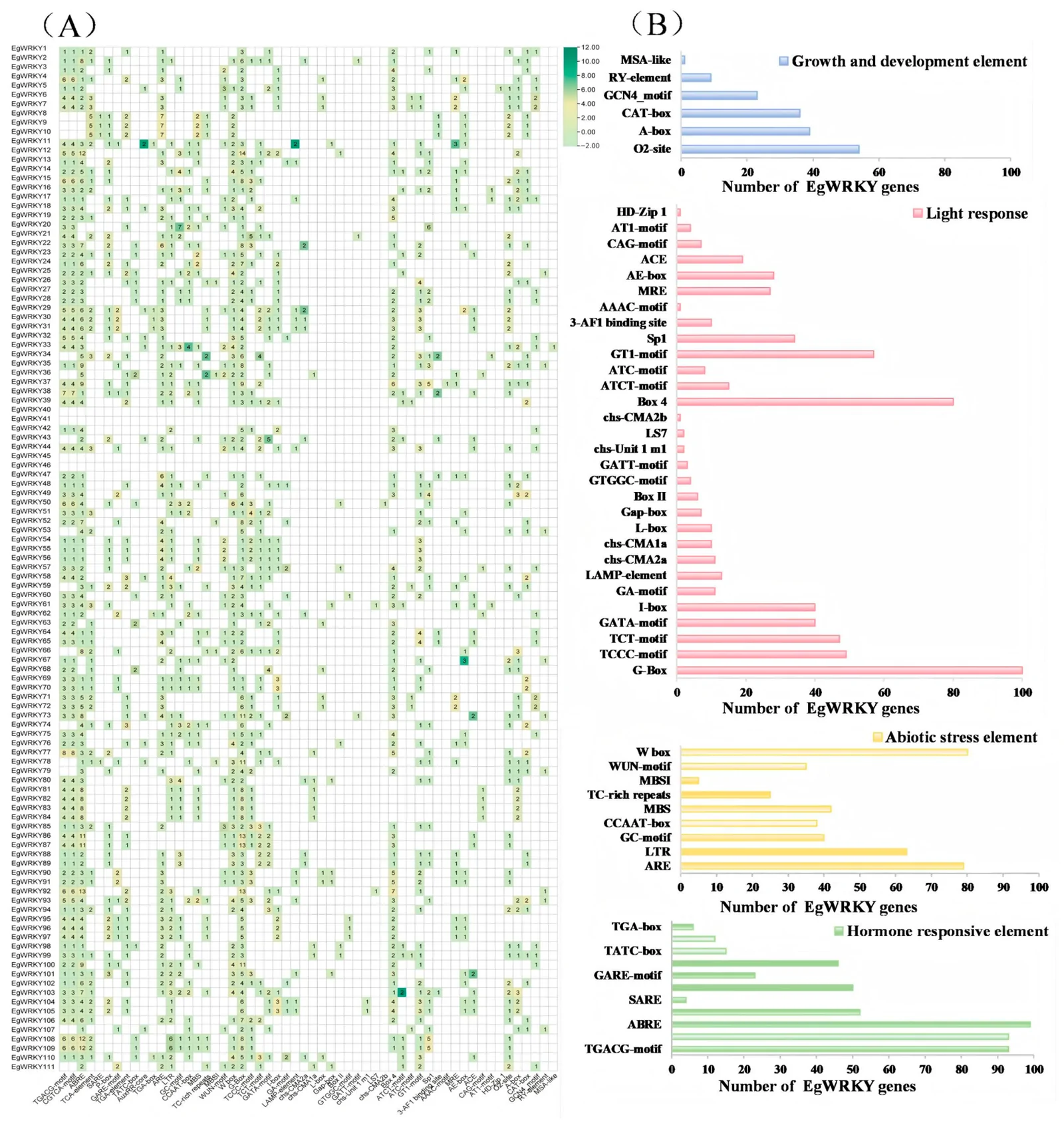
Cis-acting elements in the *EgWRKY* gene promoters. (**A**) Each element is represented by a color-coded rectangle; rectangle length is proportional to element length. Different colors indicate different functional categories. (**B**) Number of *EgWRKY* genes containing various cis-acting elements. The *x*-axis indicates gene number, the *y*-axis indicates element name, and different colors represent functional categories.

A total of 56 types of cis-acting elements were identified in the 2000 base pairs upstream of the *EgWRKY* genes, including W-box, ABRE, DRE/CRT, and MBS elements, suggesting potential involvement in hormone and stress responses (Figure 4A). Eleven plant hormone-related cis-acting elements were identified in the promoter region of the *EgWRKY* genes (TGACG-motif, CGTCA-motif, ABRE, TCA-element, SARE, P-box, GARE-motif, TGA-element, TATC-box, AuxRR-core, and TGA-box). Nine stress response-related elements were identified (ARE, LTR, GC-motif, CCAAT-box, MBS, TC-rich repeats, MBSI, WUN-motif, W-box). Thirty light response-related elements were identified. Additionally, six growth and development-related elements were identified (O2-site, A-box, CAT-box, GCN4_motif, RY-element, MSA-like). Among these, the promoter sequences of the *EgWRKY* genes contained the highest number of light response elements G-Box and MeJA response elements, with 462 G-Box and 572 MeJA response elements, respectively. The promoter regions of 80 *EgWRKY* genes contained W-box elements. Additionally, 63 *EgWRKY* genes contained the low-temperature response element LTR, 42 *EgWRKY* genes contained the drought stress response element MBS, and 25 *EgWRKY* genes contained the defense and stress response element TC-rich repeats (Figure 4B, Table S3).

### 3.4. Chromosomal Localization and Gene Duplication of the EgWRKY Genes

**Figure 5:**
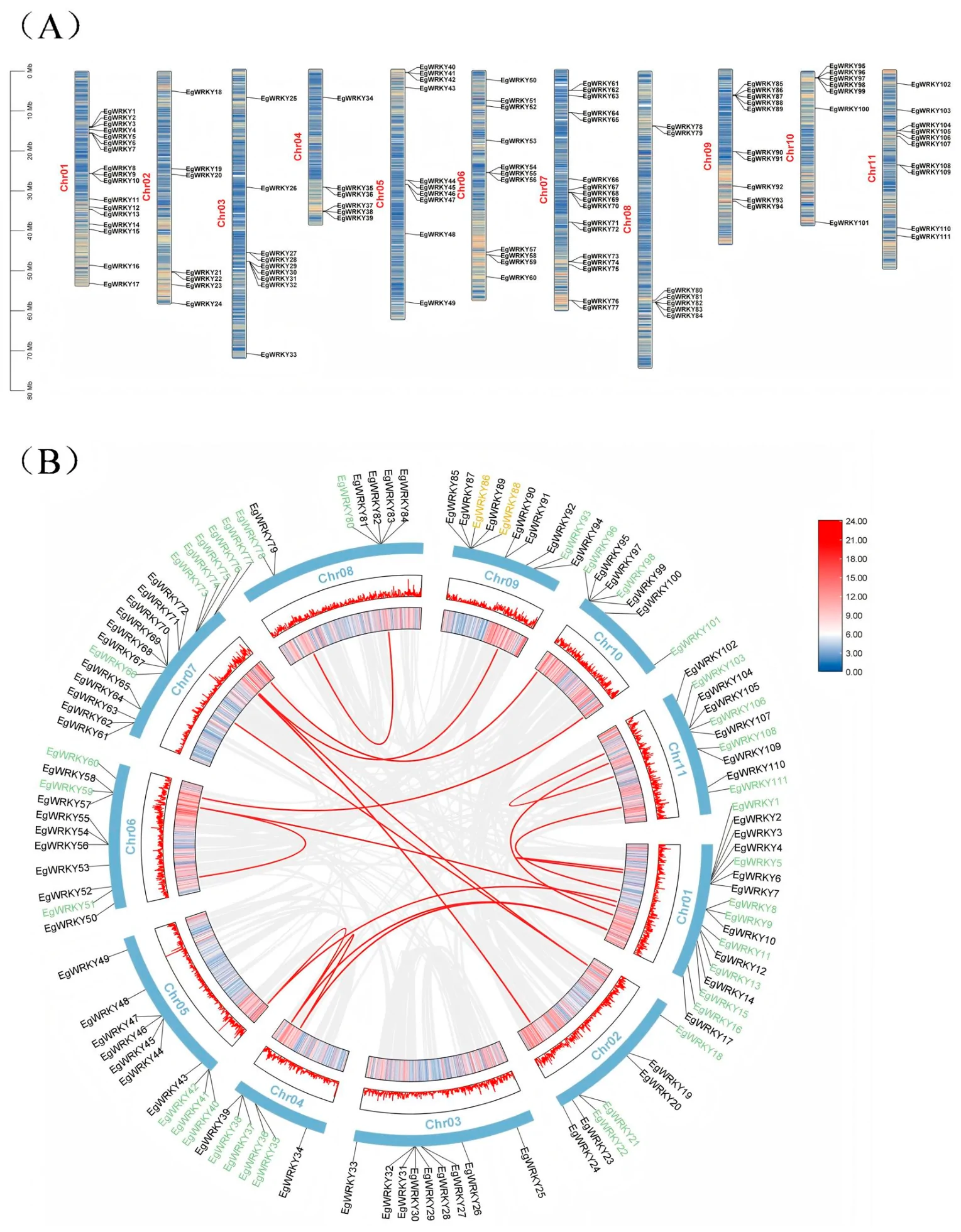
Chromosomal distribution and gene duplication map of *EgWRKY* genes. (**A**) Chromosomal distribution of *EgWRKY* genes. Colored rectangular bars represent *E. grandis* chromosomes; Chr01–Chr11 denote individual chromosomes; gene names on the right indicate approximate gene positions; the scale on the left represents chromosome length. (**B**) Chromosomal distribution of *EgWRKY* genes and their duplication relationships. Gray lines indicate collinear regions in the *E. grandis* genome, while red lines indicate duplicated WRKY gene pairs. The inner rings indicate gene density, with red indicating higher density. Yellow text denotes tandem repeats, and green text denotes segmental repeats.

The 111 *EgWRKY* genes were unevenly distributed across 11 chromosomes, with genes within the same subfamily randomly distributed across chromosomes (Figure 5A). Specifically, Chr01 contained 17 genes (*EgWRKY1*-*EgWRKY17*, 15.31%), Chr02 contained 7 genes (*EgWRKY18*-*EgWRKY24*, 6.31%), Chr03 contained 9 genes (*EgWRKY25*-*EgWRKY33*, 8.11%), Chr04 contained 6 genes (*EgWRKY34*-*EgWRKY39*, 5.41%), Chr05 contained 10 genes (*EgWRKY40*-*EgWRKY49*, 9.01%), Chr06 contained 11 genes (*EgWRKY50*-*EgWRKY60*, 9.91%), Chr07 contained 17 genes (*EgWRKY61*-*EgWRKY77*, 15.31%), Chr08 contained 7 genes (*EgWRKY78*-*EgWRKY84*, 6.31%), Chr09 contained 10 genes (*EgWRKY85*-*EgWRKY94*, 9.01%), Chr10 contained 7 genes (*EgWRKY95*-*EgWRKY101*, 6.31%), and Chr11 contained 10 genes (*EgWRKY102*-*EgWRKY111*, 9.01%).

Segmental duplication and tandem duplication events are the primary mechanisms underlying the formation and expansion of gene families. The results revealed one pair of tandemly duplicated genes (*EgWRKY86* and *EgWRKY88*) on Chr09. In addition, 48 homologous loci and 24 segmental duplication gene pairs were identified (Figure 5B). This suggested that segmental duplication events may be the primary driving force behind the formation and expansion of the EgWRKY gene family. After calculating the Ka and Ks values for the gene pairs, two gene pairs yielded undefined Ka/Ks values (NaN) because Ks values could not be calculated, likely due to insufficient alignment information. The Ka/Ks values for the remaining 23 gene pairs were below 1, indicating that these genes evolved through purifying selection, with gene sequences tending to remain stable. The Ka/Ks value for tandemly duplicated genes was 0.33, while that for segmentally duplicated genes ranged from 0.12 to 0.38. The average Ka/Ks value for tandemly duplicated gene pairs (0.33) was higher than that for segmentally duplicated gene pairs (0.23) (Table S4).

### 3.5. Analysis of the Synteny Between EgWRKY Genes and Other Species

**Figure 6:**
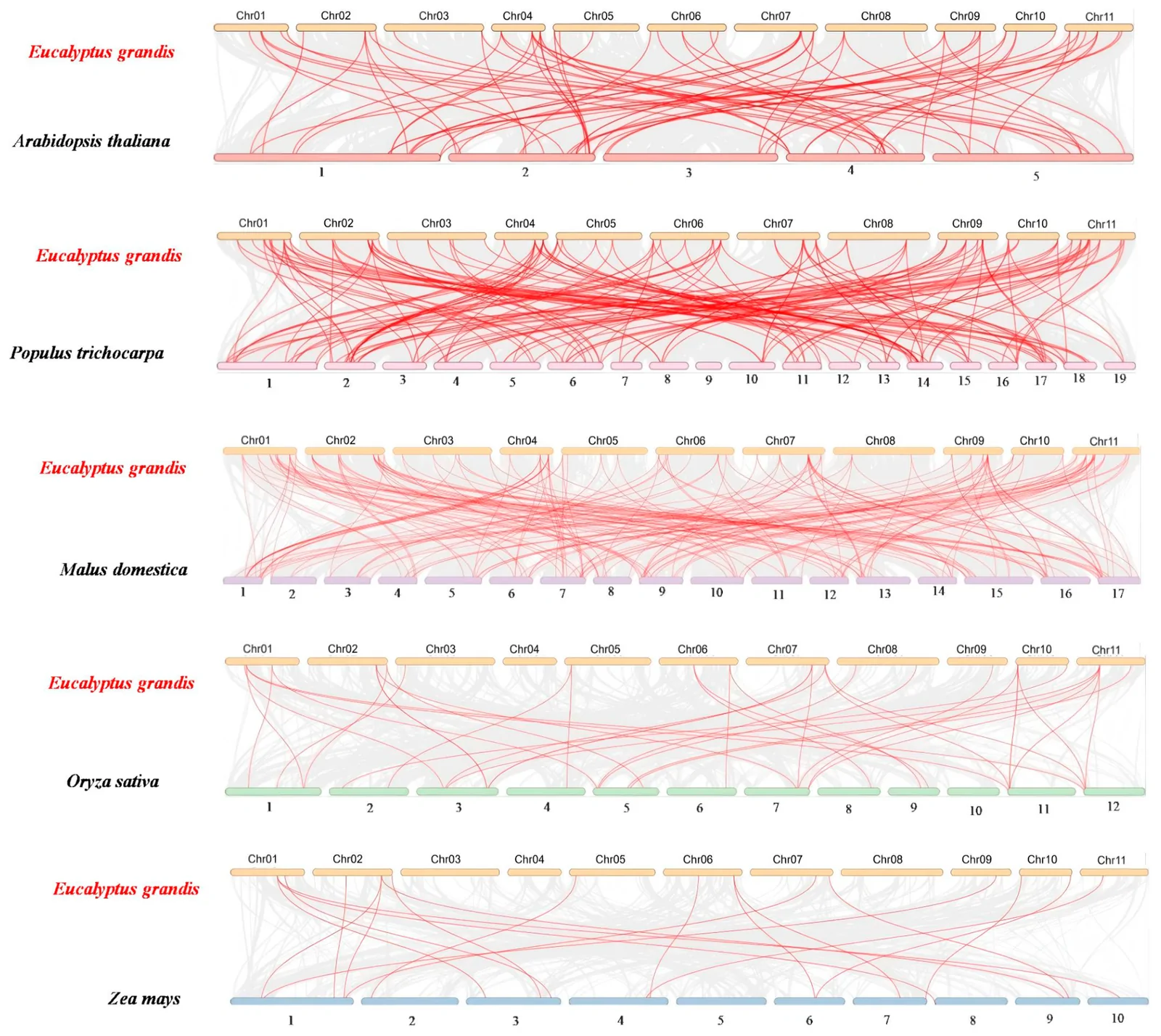
Collinearity analysis of WRKY genes between *E. grandis* and five other plant species (*O. sativa*, *Z. mays*, *A. thaliana*, *P. trichocarpa*, and *M. domestica*). Gray lines between *E. grandis* and the other species represent collinear blocks, while red lines indicate collinear pairs of WRKY genes.

To investigate the evolutionary relationships among *EgWRKY* genes and different species, we selected two monocotyledons, *O. sativa* and *Z. mays*, and three dicotyledons, *A. thaliana*, *P. trichocarpa*, and *M. domestica* to construct collinearity maps for each (Figure 6, Table S5). We found that the EgWRKY gene family exhibited relatively stronger collinearity with WRKY genes in dicotyledons than in monocots, likely reflecting their closer phylogenetic affinity. Among these, the highest collinearity was observed between *E. grandis* and *M. domestica*, with 181 colinear gene pairs. This was followed by *P. trichocarpa* with 154 pairs and *A. thaliana* with 70 colinear gene pairs. The number of colinear gene pairs between *E. grandis* and the two monocotyledons was lower, with 38 pairs in *O. sativa* and 19 pairs in *Z. mays*. Notably, *EgWRKY3*, *EgWRKY6*, *EgWRKY7*, *EgWRKY8*, *EgWRKY10*, *EgWRKY11*, *EgWRKY28*, *EgWRKY29*, *EgWRKY30*, *EgWRKY32*, *EgWRKY42*, *EgWRKY44*, *EgWRKY45*, *EgWRKY46*, *EgWRKY49*, *EgWRKY53*, *EgWRKY54*, *EgWRKY56*, *EgWRKY58*, *EgWRKY61*, *EgWRKY62*, *EgWRKY63*, *EgWRKY65*, *EgWRKY67*, *EgWRKY68*, *EgWRKY69*, *EgWRKY70*, *EgWRKY71*, *EgWRKY72*, *EgWRKY81*, *EgWRKY83*, *EgWRKY84*, *EgWRKY87*, *EgWRKY88*, *EgWRKY89*, *EgWRKY90*, *EgWRKY95*, *EgWRKY97*, *EgWRKY98*, *EgWRKY105*, and *EgWRKY109* did not share orthologs with any of the five species, suggesting that these genes may have evolved after the divergence of dicotyledons and monocotyledons.

### 3.6. Effects of AM Symbiosis on Colonization and Biomass of E. grandis Seedlings Under Drought Stress

**Figure 7:**
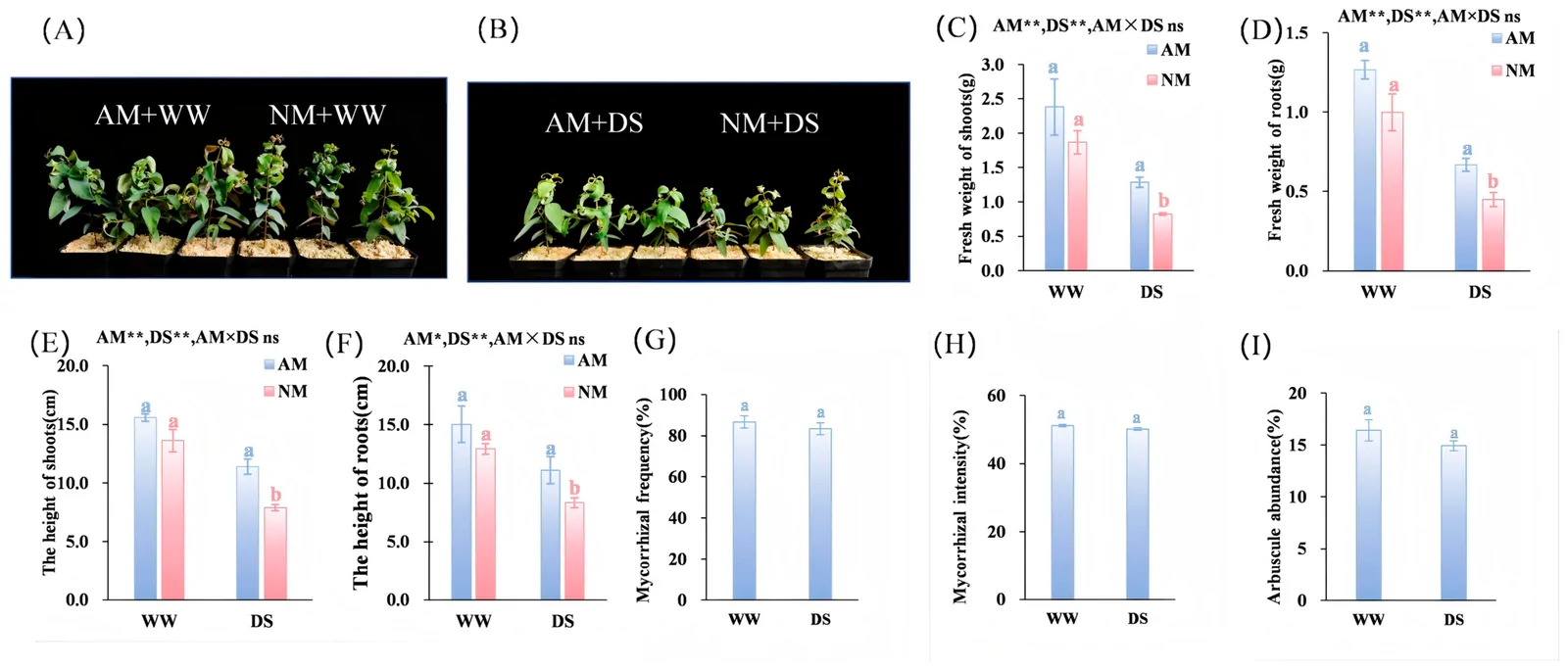
Effects of arbuscular mycorrhizal (AM) and non-mycorrhizal (NM) treatments on growth, shoot and root fresh weight, shoot height, and root length of *E. grandis* under WW (Well water) and DS (Drought stress) conditions. (**A**,**B**) Growth performances of AM-inoculated and NM *E. grandis* under WW and DS conditions. (**C**–**F**) Shoot fresh weight (**C**), root fresh weight (**D**), plant height (**E**), and root length (**F**) of *E. grandis* under different drought conditions. The data are shown as the means ± SE from three biological replicates (*n* = 3). Different letters above bars indicate statistically significant differences (*p* < 0.05) as determined by two-way ANOVA followed by Tukey’s test. Significant effect: * *p* < 0.05, ** *p* < 0.01; ns, not significant (*p* ≥ 0.05). (**G**–**I**) Total mycorrhizal frequency (**G**), mycorrhizal intensity (**H**), and arbuscule abundance (**I**) in *R. irregularis*-colonized roots were estimated after staining. The data are shown as the means ± SE from three biological replicates (*n* = 3). Different letters above bars indicate statistically significant differences (*p* < 0.05) as determined by one-way ANOVA followed by Tukey’s test.

*E. grandis* seedlings were inoculated with AM fungi and successfully colonized under WW (Well water) and DS (Drought stress) conditions. In contrast, no fungal colonization structures were observed in the root systems of non-mycorrhizal (NM) *E. grandis* seedlings. In the roots of *E. grandis* inoculated with AM fungi, typical arbuscules, vesicles, and intraradical hyphae were observed, indicating that the AM fungi established a symbiotic relationship with *E. grandis*. According to statistical analysis, there were no significant differences in the total mycorrhizal frequency, mycorrhizal intensity, and arbuscule abundance of *E. grandis* under WW and DS conditions (Figure 7G–I), indicating that drought stress did not significantly affect AM fungal colonization. The drought treatment severely affected the height, leaf color, and leaf shape of the *E. grandis* seedlings (Figure 7A,B). Under WW conditions, the plants grew normally with light green leaves. Under DS conditions, the *E. grandis* plants were stunted, with yellowish and curled leaves. However, AM seedlings were significantly taller than NM seedlings, with broader, thicker leaves that retained a light green color, indicating that AM fungal symbiosis helped alleviate the effects of drought stress on *E. grandis* seedlings. The effects of AM fungal symbiosis on *E. grandis* growth were further elucidated by measuring the biomass of AM and NM *E. grandis* under drought stress. Compared to NM *E. grandis* under DS conditions, AM *E. grandis* exhibited significantly greater biomass, plant height, and root length, indicating that AM fungal symbiosis effectively promoted the growth of *E. grandis* under drought stress (Figure 7C–F).

### 3.7. Expression of EgWRKY Genes in E. grandis Under Drought Stress with AM Symbiosis

**Figure 8:**
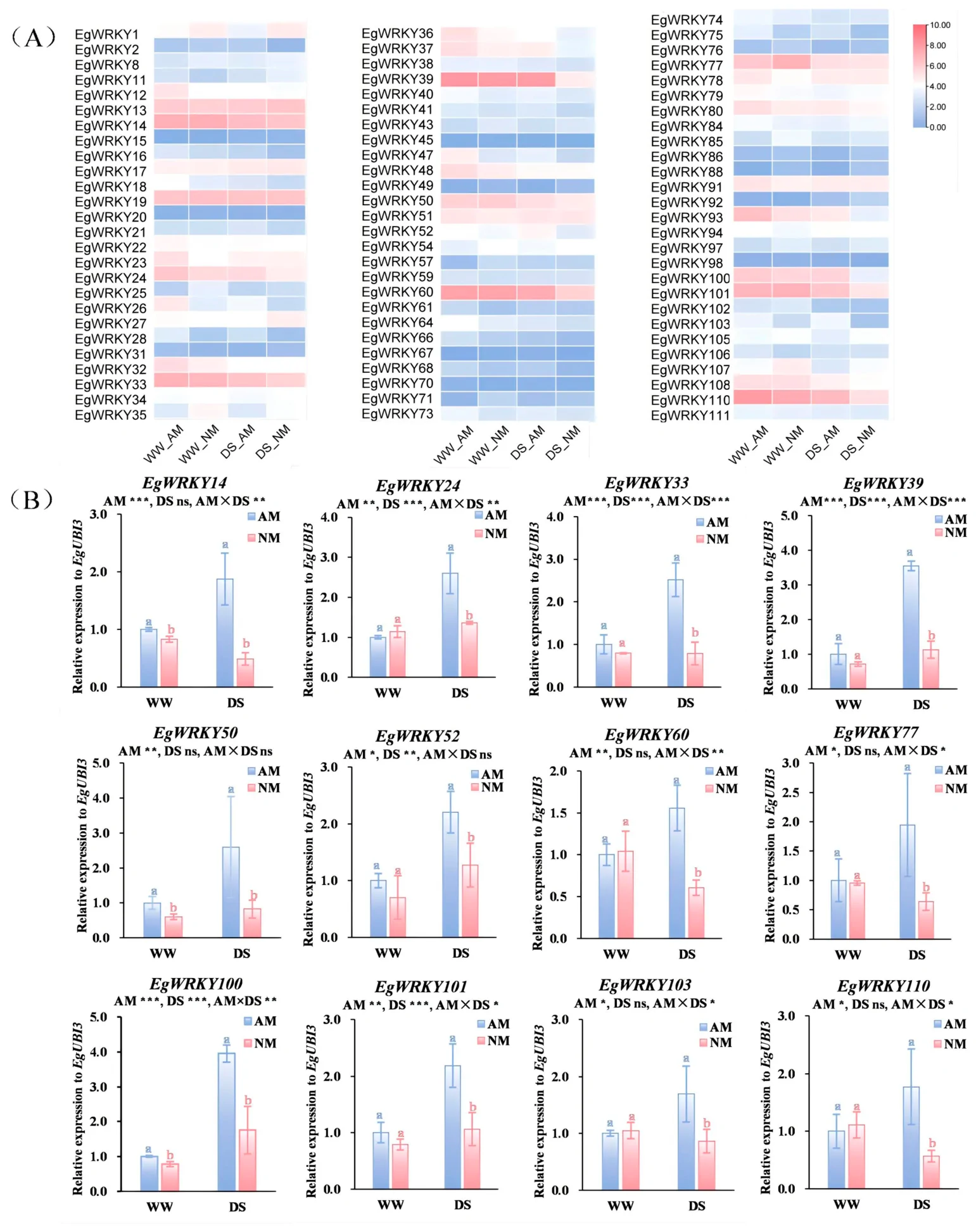
Expression of the *EgWRKY* genes under WW (Well water) and DS (Drought stress) conditions in the presence of AM fungal symbiosis. (**A**) Relative expression levels of the *EgWRKY* genes under different treatments in AM fungal symbiosis. Expression levels are expressed as log2 (FPKM + 1) and represent the average of three biological replicates. AM indicates inoculation with AM fungi; NM indicates no inoculation with AM fungi. (**B**) *EgUBI3* serves as the internal control gene for *E. grandis*. The *x*-axis represents different treatments, and the *y*-axis shows the scale of relative expression levels. Data are presented as the mean ± SE from three biological replicates (*n* = 3). Different letters above bars indicate statistically significant differences (*p* < 0.05) as determined by two-way ANOVA followed by Tukey’s test. Significant effect: * *p* < 0.05, ** *p* < 0.01, *** *p* < 0.001; ns, not significant (*p* ≥ 0.05).

Under adverse conditions, WRKY genes enhance plant tolerance to abiotic stresses, and AM fungal symbiosis also helps plants withstand stress. To analyze the expression patterns of *EgWRKY* genes under drought stress during AM fungal symbiosis, root samples from mycorrhizal and non-mycorrhizal seedlings subjected to different treatments (Well Water, WW; Drought stress, DS) were analyzed to determine RNA-seq expression levels (FPKM). The data were then visualized using TBtools-II v2.454. A total of 12 *EgWRKY* genes meeting the differential expression criteria (|log_2_ fold change| ≥ 1 and adjusted *p* value (padj) < 0.05) were identified. The results showed that under drought conditions, 12 *EgWRKY* genes (*EgWRKY14*, *EgWRKY24*, *EgWRKY33*, *EgWRKY39*, *EgWRKY50*, *EgWRKY52*, *EgWRKY60*, *EgWRKY77*, *EgWRKY100*, *EgWRKY101*, EgWRKY103, *EgWRKY110*) exhibited increased transcription levels in AM fungal symbiosis (Figure 8A). Subsequently, qRT-PCR was performed on these 12 *EgWRKY* genes (Figure 8B). The results showed that under drought conditions, AM fungal symbiosis significantly increased the expression levels of these 12 *EgWRKY* genes.

## 4. Discussion

In previous studies, Fan et al. [38] and Zhang et al. [39] identified 79 and 117 EgWRKY proteins in *E. grandis*, respectively, while 111 EgWRKY proteins were identified in this study. The differences in protein numbers may be due to differences in genome assembly versions and identification methods. Fan et al. performed BLASTP using *Arabidopsis* protein sequences against the *E. grandis* protein sequences (*Eucalyptus grandis* v2.0). Secondly, WRKY proteins were searched in the NCBI and UniProt databases. Subsequently, these proteins were verified through SMART and Pfam, and finally 79 WRKY proteins were identified. Zhang et al. identified EgWRKY proteins in the *E. grandis* protein sequences through the WRKY domain (PF03106), and subsequently verified whether the WRKY domain existed through NCBI CDD and SMART, finally identifying 117 EgWRKY proteins. In contrast, our study combined homology-based (BLASTP) and HMM-based searches using the latest genome assembly (ASM1654582v1), followed by validation with Pfam, SMART, and NCBI CDD. This strategy identified 113 candidate proteins, of which 111 were confirmed as EgWRKY proteins.

WRKY transcription factors influence plant growth, development, and stress tolerance through interactions with other signaling pathways. Studying the functions of WRKY transcription factors helps elucidate how plants balance growth and defense under adverse environmental conditions, providing a theoretical basis for breeding stress-tolerant plants. In this study, we employed various bioinformatics methods to identify 111 members of the EgWRKY gene family in the *E. grandis* genome, distributed across 11 chromosomes. We analyzed their physicochemical properties, including molecular weight (MW), theoretical isoelectric point (pI), grand average of hydropathy (GRAVY), and subcellular localization (Table S1). Significant variations among EgWRKY proteins may reflect divergent evolutionary pressures under varying environmental conditions, potentially contributing to structural diversification within the gene family. The exceptionally large size of *EgWRKY61* (1831 aa) is attributable to its chimeric domain architecture containing two WRKY domains, an NB-ARC domain, and multiple LRR repeats, suggesting potential integration of transcriptional regulation with pathogen recognition mechanisms. The isoelectric point ranged from 4.75 (*EgWRKY79*) to 11 (*EgWRKY73*). Most EgWRKY proteins had a pI < 7, indicating that this gene family is biased toward acidic amino acids. This predominance of acidic pI values is consistent with studies on dicotyledons but contrasts with those on monocotyledons [61,62]. The average GRAVY score (<0) suggests that EgWRKY proteins are hydrophilic. Subcellular localization analysis using WoLF PSORT predicted that EgWRKY proteins were localized to the nucleus, chloroplasts, endoplasmic reticulum, cytoplasm, peroxisomes, and Golgi apparatus (Table S1). Notably, the majority of EgWRKY proteins were predicted to localize to the nucleus, consistent with their primary roles as transcription factors.

The EgWRKY gene family can be classified into three major groups (I, II, and III) and five subgroups (IIa, IIb, IIc, IId, and IIe), which is consistent with previous findings and indicates relative stability during evolution [63]. Four major WRKY lineages have been identified in angiosperms: Clade I + Clade IIc, Clade IIa + Clade IIb, Clade IId + Clade IIe, and Clade III, accurately reflecting the evolutionary history of the WRKY family, a pattern also validated in *E. grandis* [64]. The expansion of Group III (24 members, the largest subgroup) in the EgWRKY gene family may reflect the evolutionary adaptation of *E. grandis* to environmental stresses. Group III WRKY genes are frequently associated with stress responses, and their expansion in *E. grandis* suggests a potential selective advantage in coping with abiotic stresses such as drought.

Further sequence alignment of EgWRKY and AtWRKY proteins revealed that Group I contains two WRKY domains located at the C- and N-termini, whereas Groups II and III contain only a single WRKY domain, consistent with findings from other studies (Figure 2) [3]. The C-terminal domain of EgWRKY Group I members is more conserved than the N-terminal domain, and Groups II and III arise from deletions at the N-terminal end of Group I, consistent with previous findings [65]. Differences in conserved sequences exist between subgroups, and WRKY domains within the same subgroup are not entirely identical, enabling EgWRKY proteins to perform distinct functions during plant growth and development. Most EgWRKY proteins (93/111) contain highly conserved WRKY domains; however, the *EgWRKY49* protein exhibits a domain deletion, which may alter its DNA-binding properties or regulatory capacity. Additionally, various heptapeptide sequence mutations (WKKHGQK, WRKYGKK, WRKYGQR, WRKYAQE, WRKYDQK, WTMRQR, WRKYGSK) were identified in 17 proteins. Similar variations have been reported in other species, including three Group IIc members in pineapple, where such variants may affect target gene interactions and require further functional validation [1]. In apple, seven *MdWRKY* genes carry similar heptapeptide alterations, a phenomenon also noted in *Arabidopsis*, *Cucumis sativus*, and barley [66,67]. These observations suggest that the 17 *EgWRKY* variants identified here may possess modified DNA-binding affinities or target specificities, and their biological functions warrant further experimental investigation.

The gene structures and conserved protein motifs of the EgWRKY gene family in *E. grandis* are highly conserved (Figure 3). Ten conserved motifs were identified among the 111 *EgWRKY* genes. The distribution of conserved motifs, exons, and introns among *EgWRKY* genes within the same group or subgroup shows a high degree of similarity, indicating that members of the same group or subgroup are highly conserved and may have similar evolutionary origins and biological functions, consistent with previous findings [68,69]. Motif 8 was found only in members of Group I, motif 7 was found only in members of Subgroups IIa and IIb, while motif 9 was found only in members of Group III, indicating distinct conservation patterns among EgWRKY subgroups. Whether these subgroup-specific motifs contribute to functional divergence in transcriptional regulation, protein–protein interactions, or stress responses remains unknown and requires experimental validation. The gene promoter is a DNA sequence located upstream of the coding region and contains multiple cis-acting elements, which serve as protein-specific binding sites involved in transcription initiation and regulation [70]. Cis-acting elements are important regulatory elements that control gene expression and play a crucial role in responding to internal and external environmental signals. Promoter analyses have identified a large number of abiotic stress response cis-acting elements in the promoter regions of WRKY genes across multiple species, suggesting that most plant WRKY genes likely play a role in controlling responses to abiotic stress [71]. In this study, we predicted cis-acting elements for 111 *EgWRKY* genes based on their promoter sequences (Figure 4, Table S3) and found that *EgWRKY* genes contain various response elements, such as those associated with abiotic stress and plant hormones. Their promoters contain the low-temperature response element LTR, the drought stress response element MBS, and TC-rich repeats associated with defense and stress responses. Based on these findings, we suggest that *EgWRKY* genes may be involved in plant growth and development and play important roles in abiotic stress responses. The presence of W-box elements in the promoters of 80 *EgWRKY* genes predicts potential binding sites for WRKY transcription factors. However, promoter analysis alone does not demonstrate actual regulatory interactions, and experimental validation would be required to confirm cross-regulation or autoregulation.

The varying numbers of WRKY gene family members across different species may be related to tandem and segmental duplication of genes during evolution. These processes drive the expansion of the WRKY gene family, while gene duplication and divergence lead to the emergence of distinct family members [72,73]. *EgWRKY* genes are unevenly distributed across 11 chromosomes. One tandem duplication pair and 24 segmental duplication pairs were identified among the *EgWRKY* genes, involving 50 *EgWRKY* genes, accounting for 45.04% of the EgWRKY family members, indicating that segmental duplication events play a role in the evolution of the EgWRKY gene family (Figure 5B and Table S4). Generally, a Ka/Ks ratio of 1 indicates neutral selection; a Ka/Ks ratio > 1 indicates adaptive evolution driven by positive selection, while a Ka/Ks ratio < 1 indicates negative selection or purifying selection [74,75]. The results of this study showed that the EgWRKY gene family is highly conserved during evolution, consistent with previous findings [76]. In *G. max*, segmental duplication has driven WRKY family expansion, with positive selection as a key force behind duplicated gene divergence, critical for plant stress responses over evolutionary time [77]. Across 42 angiosperms, gene family expansions are integral to context-dependent regulation of species interactions; mycorrhizal plants with expanded gene families show up to 200% more context-dependent gene expression and doubled genetic variation linked to mycorrhizal fitness benefits. These expansions arise primarily from tandem duplications at >2-fold genome-wide rates, continuously supplying genetic variation for fine-tuned context dependency throughout plant evolution. Thus, widespread gene duplications provide molecular flexibility for plant–microbial interactions under changing environments [78]. We hypothesize that duplicated *EgWRKY* genes may have similarly diverged to coordinate drought responses and AM fungal symbiosis in *E. grandis*, awaiting experimental validation. Furthermore, we analyzed the collinearity of WRKY genes between *E. grandis* and three dicotyledonous plants (*A. thaliana*, *P. trichocarpa*, *M. domestica*) as well as two monocotyledonous plants (*O. sativa*, *Z. mays*). The EgWRKY gene family exhibited greater collinearity with dicotyledons than with monocotyledons, consistent with its closer evolutionary relationship with dicotyledonous WRKYs (Figure 6, Table S5). Some *EgWRKY* genes showed no syntenic relationships with the five plant species, possibly due to gene loss, genome rearrangements, or annotation limitations. We suggest that members of the EgWRKY gene family may have undergone lineage-specific expansion, potentially contributing to the evolution of *E. grandis* [42].

WRKY transcription factors play a significant role in plant drought response. While root-focused analysis is justified by the root-localized nature of AM symbiosis, we recognize that excluding leaf and stem tissues limits our ability to assess systemic WRKY-mediated drought responses. This study demonstrated that AM fungal symbiosis can form a symbiotic relationship with *E. grandis* and significantly increase the biomass, plant height, and root length of *E. grandis* seedlings (Figure 7). This growth-promoting effect is consistent with other studies [32]. The results showed that under drought conditions, 12 *EgWRKY* genes (*EgWRKY14*, *EgWRKY24*, *EgWRKY33*, *EgWRKY39*, *EgWRKY50*, *EgWRKY52*, *EgWRKY60*, *EgWRKY77*, *EgWRKY100*, *EgWRKY101*, *EgWRKY103*, *EgWRKY110*) exhibited increased transcript levels following AM fungal inoculation, indicating that they play a significant role in responding to drought stress (Figure 8). The 12 drought/AM-responsive *EgWRKY* candidates represent regulatory nodes integrating AM fungal symbiotic signaling with drought adaptation in *E. grandis*. Group IId candidates (*EgWRKY14*, *EgWRKY33*, and *EgWRKY50*) are homologous to *AtWRKY11*/*AtWRKY17*, which form WRKY-OBE complexes to suppress stress genes under non-stress conditions, thereby maintaining normal growth; drought-induced down-regulation of these repressors activates stress-responsive genes to enhance tolerance [14]. Notably, *EgWRKY33* shows the highest homology to *AtWRKY11* and *AtWRKY17*. We hypothesize that AM fungal symbiosis modulates these *EgWRKY* genes to fine-tune the growth–defense trade-off under combined stress. Group III candidates (*EgWRKY39*, *EgWRKY77*, and *EgWRKY100*) cluster with *Arabidopsis* Group III WRKYs, among which *AtWRKY46*/*AtWRKY54*/*AtWRKY70* regulate drought via BR signaling [15], *AtWRKY63* acts downstream of *ABI1*/*ABI2*/*ABI5* in the ABA cascade [79], and *AtWRKY53* binds the QQS promoter to reduce hydrogen peroxide and enhance sugar metabolism [11]. Notably, *EgWRKY39* and *EgWRKY100* show the highest homology to *AtWRKY53*. Both genes also contain MBS elements in their promoters, raising the possibility that they function as ABA-dependent regulatory hubs in AM–drought crosstalk. Given these structural and phylogenetic features, *EgWRKY39* and *EgWRKY100* represent particularly promising targets for dissecting ABA-dependent AM–drought molecular mechanisms in *E. grandis*.

## 5. Conclusions

*E. grandis* is an important fast-growing industrial timber species widely used in various fields, including structural lumber and engineered wood products. In this study, 111 *EgWRKY* genes were identified from the *E. grandis* genome. Bioinformatics analyses and gene expression profiling identified *EgWRKY* genes as candidate regulators potentially involved in plant growth and development as well as drought stress responses. The results showed that the physicochemical properties of the EgWRKY gene family varied significantly, while gene structures and conserved motifs were highly conserved. These 111 *EgWRKY* genes were unevenly distributed across 11 chromosomes, and were associated with one tandem duplication event and 24 segmental duplication events. Collinearity analysis showed the strongest syntenic relationships with dicotyledons. Furthermore, AM fungal symbiosis with *E. grandis* significantly increased the biomass, plant height, and root length of *E. grandis* seedlings. qRT-PCR analysis revealed that 12 *EgWRKY* genes (*EgWRKY14*, *EgWRKY24*, *EgWRKY33*, *EgWRKY39*, *EgWRKY50*, *EgWRKY52*, *EgWRKY60*, *EgWRKY77*, *EgWRKY100*, *EgWRKY101*, *EgWRKY103*, *EgWRKY110*) showed transcriptional responsiveness to drought stress under AM fungal symbiosis, suggesting that they may function as candidate regulators involved in drought tolerance. However, RNA-seq and qRT-PCR analyses demonstrate transcriptional responsiveness but do not establish regulatory function; the precise functional roles of these candidate genes await validation through genetic approaches such as overexpression, knockout, or gene silencing experiments. These results provide insights for further exploration of the functions of *EgWRKY* genes in the growth, development, and drought stress response of *E. grandis*.

## Data Availability

Data will be made available on request.

## Supplementary Materials

The following supporting information can be downloaded at: https://www.mdpi.com/article/10.3390/microorganisms14081626/s1. Figure S1: Domain predictions for the EgWRKY gene family; Figure S2: Sequence logos for conserved motifs; Table S1: Molecular weight, theoretical isoelectric point, hydrophilicity, and subcellular localization of EgWRKY proteins; Table S2: Analysis of conserved motifs in the EgWRKY proteins; Table S3: Cis-acting elements in the *EgWRKY* genes promoter region; Table S4: Ka/Ks analysis of *EgWRKY* genes in *E. grandis*; Table S5: Genetic linkage pairs between *E. grandis* and five other plant species; Table S6: Primers used in the paper.
