# Supplementary material for: Genome-Wide Identification and Expression Analysis of the WRKY Gene Family in Eucalyptus grandis Under Drought Stress During Arbuscular Mycorrhizal Symbiosis
Source: Microorganisms. 2026 Jul 25;14(8):1626. doi: 10.3390/microorganisms14081626 (PMC13515241; doi:10.3390/microorganisms14081626)
Supplement: Supplementary file 1 [file microorganisms-14-01626-s001.zip › microorganisms-4417966-supplementary.pdf]

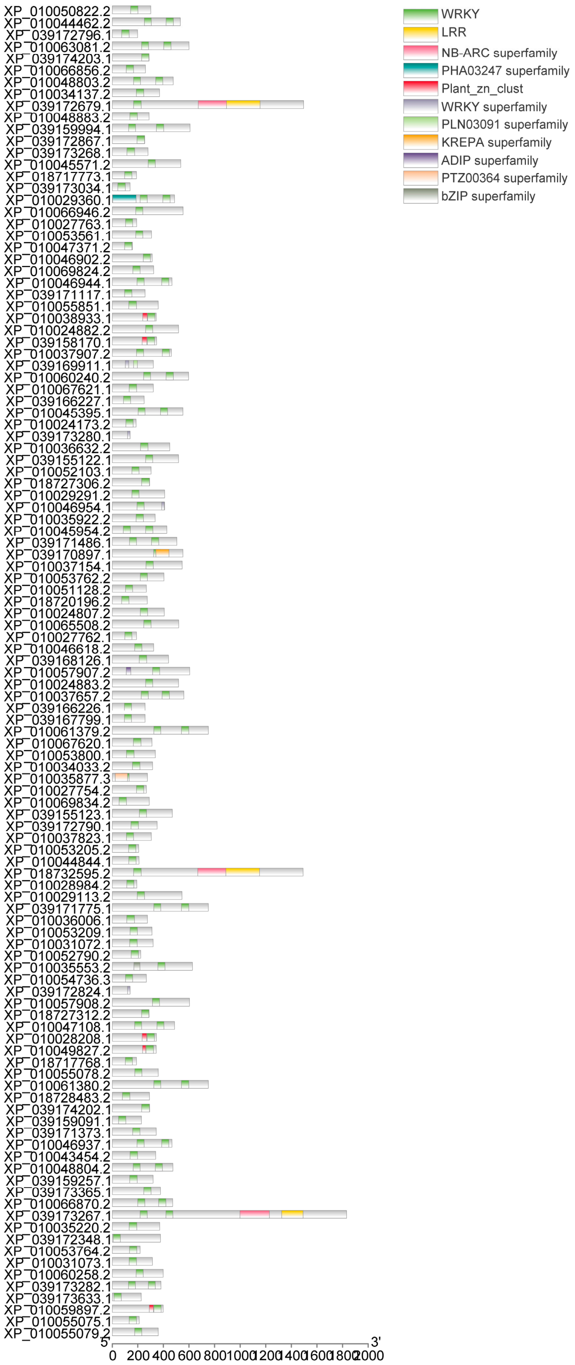

Figure S1. Domain predictions for the EgWRKY gene family. The colored boxes represent different domains.

Table S1. Molecular weight, theoretical isoelectric point, hydrophilicity, and subcellular localization of EgWRKY proteins.

Gene name	Gene ID	Chr	Length (aa)	MW (Da)	PI	Gravy	Subcellular Localization (WoLF PSORT)
EgWRKY1	XM_010045152.3	Chr01	339	37345.00	5.44	-0.819	Nucleus
EgWRKY2	XM_039316856.1	Chr01	351	38484.54	9.11	-0.556	Nucleus
EgWRKY3	XM_039316862.1	Chr01	198	22267.80	8.57	-0.786	Nucleus
EgWRKY4	XM_039316933.1	Chr01	253	28691.82	6.07	-0.721	Nucleus
EgWRKY5	XM_039317100.1	Chr01	139	16144.02	8.52	-1.124	Nucleus
EgWRKY6	XM_018871767.2	Chr01	288	32642.29	5.26	-0.686	Nucleus
EgWRKY7	XM_018871761.2	Chr01	292	33034.77	5.26	-0.648	Nucleus
EgWRKY8	XM_010048635.3	Chr01	467	51271.73	8.88	-0.948	Nucleus
EgWRKY9	XM_010048642.3	Chr01	467	51271.73	8.88	-0.948	Nucleus
EgWRKY10	XM_010048652.3	Chr01	409	44692.47	8.65	-0.915	Nucleus
EgWRKY11	XM_010025871.3	Chr01	186	21234.54	9.12	-1.035	Peroxisome
EgWRKY12	XM_010047269.3	Chr01	535	57499.43	6.38	-0.603	Nucleus
EgWRKY13	XM_010047652.3	Chr01	427	46189.94	6.38	-1.003	Nucleus
EgWRKY14	XM_010048600.3	Chr01	312	34249.73	10.12	-0.698	Nucleus
EgWRKY15	XM_010055259.3	Chr01	307	34742.45	7.65	-0.999	Nucleus
EgWRKY16	XM_010061956.3	Chr01	398	42451.95	5.29	-0.646	Nucleus
EgWRKY17	XM_010048806.3	Chr01	486	52523.28	8.84	-0.827	Nucleus
EgWRKY18	XM_010030811.3	Chr02	545	58935.36	6.92	-0.684	Nucleus
EgWRKY19	XM_010040631.3	Chr02	343	38380.47	9.64	-0.775	Nucleus
EgWRKY20	XM_039304060.1	Chr02	608	66515.53	5.47	-0.783	Nucleus
EgWRKY21	XM_010048316.3	Chr02	324	34396.83	10.06	-0.873	Nucleus
EgWRKY22	XM_010046160.3	Chr02	532	57412.09	6.96	-0.730	Nucleus
EgWRKY23	XM_010046542.3	Chr02	210	23489.46	8.94	-0.719	Nucleus
EgWRKY24	XM_010047093.3	Chr02	552	60279.88	6.52	-0.910	Nucleus
EgWRKY25	XM_010049069.3	Chr03	158	18159.96	5.28	-1.184	Nucleus
EgWRKY26	XM_010052520.3	Chr03	301	33700.10	8.09	-0.667	Nucleus
EgWRKY27	XM_010050501.3	Chr03	477	50505.60	9.20	-0.723	Nucleus
EgWRKY28	XM_010050502.3	Chr03	474	50165.13	9.15	-0.738	Nucleus
EgWRKY29	XM_039310293.1	Chr03	250	28380.34	9.40	-0.724	Nucleus
EgWRKY30	XM_039310292.1	Chr03	257	29251.36	9.23	-0.717	Nucleus
EgWRKY31	XM_010052826.3	Chr03	266	30233.39	9.14	-0.725	Nucleus
EgWRKY32	XM_010050581.3	Chr03	288	32388.51	8.73	-0.668	Nucleus
EgWRKY33	XM_010051525.3	Chr03	343	36802.74	9.86	-0.527	Nucleus
EgWRKY34	XM_010053801.3	Chr04	304	33909.06	6.73	-1.060	Nucleus
EgWRKY35	XM_010054903.3	Chr04	207	23032.32	9.51	-0.609	Nucleus
EgWRKY36	XM_010054907.3	Chr04	311	34713.35	5.29	-0.793	Nucleus

EgWRKY37	XM_010055460.3	Chr04	403	43098.76	6.48	-0.473	Chloroplast
EgWRKY38	XM_010055462.3	Chr04	218	24280.37	8.89	-0.736	Nucleus
EgWRKY39	XM_010055498.3	Chr04	337	37596.97	5.36	-0.578	Nucleus
EgWRKY40	XM_010056773.3	Chr05	211	23384.93	9.43	-0.426	Peroxisome
EgWRKY41	XM_010056776.3	Chr05	360	38298.81	5.31	-0.669	Nucleus
EgWRKY42	XM_010056777.3	Chr05	359	38211.73	5.31	-0.669	Nucleus
EgWRKY43	XM_010054488.3	Chr05	222	24667.17	8.21	-0.836	Cytoplasm
EgWRKY44	XM_010056434.3	Chr05	266	30159.12	8.56	-0.707	Nucleus
EgWRKY45	XM_039311865.1	Chr05	257	29177.09	8.77	-0.699	Nucleus
EgWRKY46	XM_039312192.1	Chr05	440	47759.91	5.44	-0.731	Nucleus
EgWRKY47	XM_010038330.3	Chr05	450	48954.33	5.44	-0.714	Nucleus
EgWRKY48	XM_018872938.2	Chr05	291	32127.67	5.19	-0.867	Nucleus
EgWRKY49	XM_039313977.1	Chr05	321	35856.58	5.27	-0.747	Nucleus
EgWRKY50	XM_010061595.3	Chr06	398	42666.01	9.69	-0.606	Nucleus
EgWRKY51	XM_010061938.3	Chr06	597	64225.42	6.09	-0.622	Nucleus
EgWRKY52	XM_039314963.1	Chr06	553	59821.92	5.96	-0.510	Endoplasmic Reticulum
EgWRKY53	XM_039315183.1	Chr06	256	28711.53	9.20	-0.717	Nucleus
EgWRKY54	XM_010063077.3	Chr06	751	81118.96	5.79	-0.811	Nucleus
EgWRKY55	XM_039315841.1	Chr06	751	81118.96	5.79	-0.811	Nucleus
EgWRKY56	XM_010063078.3	Chr06	751	81118.96	5.79	-0.811	Nucleus
EgWRKY57	XM_010039521.3	Chr06	305	33648.52	5.40	-0.728	Nucleus
EgWRKY58	XM_039315552.1	Chr06	505	55556.66	7.67	-0.767	Nucleus
EgWRKY59	XM_010064779.3	Chr06	600	65466.98	8.71	-0.715	Nucleus
EgWRKY60	XM_039315439.1	Chr06	343	37976.11	8.02	-0.917	Nucleus
EgWRKY61	XM_039317333.1	Chr07	1831	204607.10	5.41	-0.461	Chloroplast
EgWRKY62	XM_039317334.1	Chr07	279	31506.42	8.90	-0.734	Golgi Apparatus
EgWRKY63	XM_039317348.1	Chr07	381	41532.88	9.75	-0.785	Nucleus
EgWRKY64	XM_010067206.3	Chr07	520	57026.99	5.58	-0.823	Nucleus
EgWRKY65	XM_039317431.1	Chr07	376	41669.11	5.11	-0.850	Nucleus
EgWRKY66	XM_010038852.3	Chr07	546	59737.22	6.45	-0.761	Nucleus
EgWRKY67	XM_039317699.1	Chr07	227	25537.69	6.50	-0.604	Golgi Apparatus
EgWRKY68	XM_039316414.1	Chr07	376	42083.23	6.33	-0.730	Nucleus
EgWRKY69	XM_039316745.1	Chr07	1496	168705.47	5.45	-0.414	Chloroplast
EgWRKY70	XM_018877050.2	Chr07	1491	168153.78	5.41	-0.411	Chloroplast
EgWRKY71	XM_039318269.1	Chr07	288	32608.27	5.26	-0.683	Nucleus
EgWRKY72	XM_039318268.1	Chr07	292	33000.76	5.26	-0.645	Nucleus
EgWRKY73	XM_010068554.3	Chr07	260	28683.36	11.00	-0.954	Nucleus
EgWRKY74	XM_010068568.3	Chr07	473	51487.40	6.74	-0.726	Nucleus
EgWRKY75	XM_010068644.3	Chr07	554	60312.48	7.03	-0.601	Nucleus
EgWRKY76	XM_010069318.3	Chr07	311	34238.62	7.61	-0.557	Nucleus
EgWRKY77	XM_010069319.3	Chr07	321	36166.04	5.28	-0.901	Nucleus
EgWRKY78	XM_010071522.3	Chr08	324	35578.49	6.32	-0.737	Nucleus

EgWRKY79	XM_010071532.3	Chr08	289	32306.49	4.75	-0.930	Nucleus
EgWRKY80	XM_010026505.3	Chr08	406	45260.75	6.02	-0.975	Nucleus
EgWRKY81	XM_039299188.1	Chr08	518	56089.47	6.31	-0.583	Nucleus
EgWRKY82	XM_010026581.2	Chr08	518	56089.47	6.31	-0.583	Nucleus
EgWRKY83	XM_010026580.2	Chr08	518	56089.47	6.31	-0.583	Nucleus
EgWRKY84	XM_039299189.1	Chr08	469	50391.69	5.91	-0.614	Nucleus
EgWRKY85	XM_010029452.3	Chr09	267	29957.59	8.94	-0.704	Nucleus
EgWRKY86	XM_010029460.3	Chr09	190	21417.64	7.67	-1.062	Nucleus
EgWRKY87	XM_018862228.2	Chr09	189	21261.45	6.84	-1.043	Nucleus
EgWRKY88	XM_010029461.3	Chr09	191	21792.04	5.61	-0.876	Nucleus
EgWRKY89	XM_018862223.2	Chr09	190	21635.85	5.45	-0.857	Nucleus
EgWRKY90	XM_010029906.3	Chr09	345	37407.42	9.61	-0.485	Nucleus
EgWRKY91	XM_039302236.1	Chr09	346	37494.50	9.61	-0.486	Nucleus
EgWRKY92	XM_010030682.3	Chr09	192	21453.28	9.74	-0.756	Nucleus
EgWRKY93	XM_010030989.3	Chr09	409	44530.65	6.11	-0.720	Nucleus
EgWRKY94	XM_010031058.3	Chr09	487	52689.82	7.65	-0.699	Nucleus
EgWRKY95	XM_010032771.3	Chr10	314	34439.222	5.54	-0.624	Nucleus
EgWRKY96	XM_039303323.1	Chr10	319	35070.88	5.45	-0.653	Nucleus
EgWRKY97	XM_010032770.3	Chr10	319	35070.88	5.45	-0.653	Nucleus
EgWRKY98	XM_010035835.3	Chr10	369	40254.86	6.35	-0.704	Nucleus
EgWRKY99	XM_039303157.1	Chr10	228	24965.75	8.97	-0.886	Nucleus
EgWRKY100	XM_010057549.3	Chr10	360	40366.50	5.58	-0.753	Nucleus
EgWRKY101	XM_010035731.3	Chr10	315	34845.07	7.63	-0.742	Nucleus
EgWRKY102	XM_010036918.3	Chr11	371	40489.64	5.55	-0.657	Nucleus
EgWRKY103	XM_010037251.3	Chr11	627	67267.87	6.64	-0.655	Nucleus
EgWRKY104	XM_010037575.3	Chr11	275	29795.80	5.47	-0.761	Nucleus
EgWRKY105	XM_018864651.2	Chr11	274	29708.72	5.47	-0.761	Nucleus
EgWRKY106	XM_010037620.3	Chr11	335	37418.39	6.51	-0.925	Nucleus
EgWRKY107	XM_010037704.3	Chr11	275	20765.10	7.68	-0.954	Nucleus
EgWRKY108	XM_010059605.3	Chr11	605	64404.00	6.44	-0.625	Nucleus
EgWRKY109	XM_010059606.3	Chr11	604	64275.87	6.44	-0.620	Nucleus
EgWRKY110	XM_010039355.3	Chr11	559	61246.24	6.91	-0.871	Nucleus
EgWRKY111	XM_010039605.3	Chr11	462	50296.12	9.41	-0.891	Nucleus

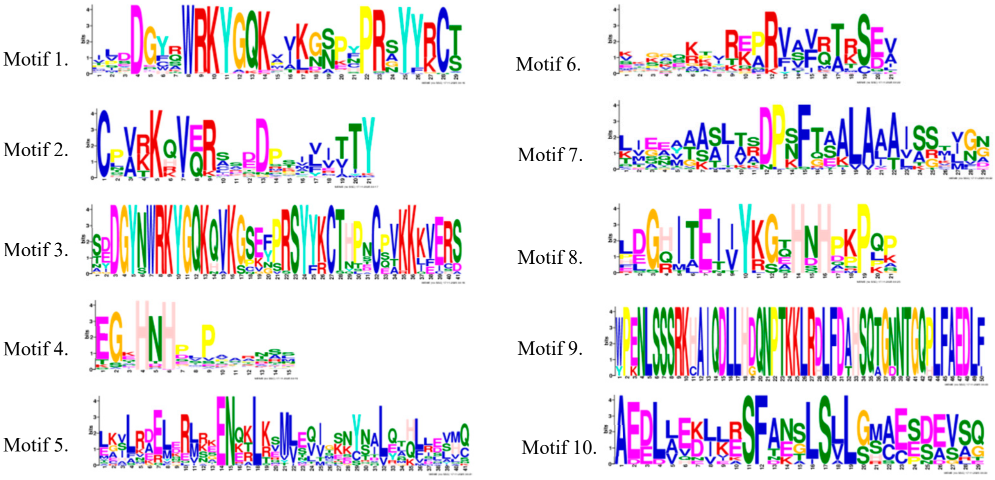

Figure S2. Sequence logos for conserved motifs. The y-axis (in bits) represents the overall height of the stack, indicating sequence conservation at that position, while the height of each symbol within the stack indicates the relative frequency of each amino acid at that position.

Table S2. Analysis of conserved motifs in the EgWRKY proteins.

Motif	E-value	Sites	Width	Multilevel consensus sequence
**1**	2.0e-2430	110	29	ILDDGYRWRKYGQKVVKGSPYPRSYYRCT
**2**	3.6e-1306	111	21	CPVRKQVZRSSDDPSILITTY
**3**	1.5e-595	20	41	SDDGYNWRKYGQKQVKGSEFPRSYYKCTHPNCPVKKKVERS
**4**	3.1e-319	82	15	EGKHNHPLPAARNSS
**5**	7.9e-259	21	41	LKVLRDELERLRKENQKLKSMLEQIGKNYNALZTHLLEVMQ
**6**	5.8e-232	46	21	KKGGQKKVREPRVAVRTRSEV
**7**	8.5e-206	24	29	LIEEAAASJTRDPNFTAALAAAISSIVGN
**8**	7.4e-187	19	21	LDGHITEIIYKGQHNHPKPQP
**9**	1.1e-168	6	50	WPENLSSSRKHAIQDLLHDQNPTKKLRDLFDAHSQTGNNTGQPLFA EDLF
**10**	5.7e-097	12	29	AEDLLEDJLESFANSLSLLGMAESDEVSQ

Table S3. Cis-acting elements in the *EgWRKY* genes promoter region.

Cis-acting elements	Cis-acting elements number	Function	Functional classification	Number of EgWRKY genes
TGACG-motif	286	cis-acting regulatory element involved in the MeJA-responsiveness	Hormone responsive element	93
CGTCA-motif	286	cis-acting regulatory element involved in the MeJA-responsiveness	Hormone responsive element	93
ABRE	425	cis-acting element involved in the abscisic acid responsiveness	Hormone responsive element	99
TCA-element	95	cis-acting element involved in salicylic acid responsiveness	Hormone responsive element	52
SARE	4	cis-acting element involved in salicylic acid responsiveness	Hormone responsive element	4
P-box	66	gibberellin-responsive element	Hormone responsive element	50
GARE-motif	31	gibberellin-responsive element	Hormone responsive element	23
TGA-element	63	auxin-responsive element	Hormone responsive element	46
TATC-box	18	cis-acting element involved in gibberellin-responsiveness	Hormone responsive element	15
AuxRR-core	13	cis-acting regulatory element involved in auxin responsiveness	Hormone responsive element	12
TGA-box	6	part of an auxin-responsive element	Hormone responsive element	6
ARE	183	cis-acting regulatory element essential for the anaerobic induction	Abiotic stress element	79
LTR	97	cis-acting element involved in low-temperature responsiveness	Abiotic stress element	63
GC-motif	70	enhancer-like element involved in anoxic specific inducibility	Abiotic stress element	40
CCAAT-box	50	MYBHv1 binding siteMYBHv1	Abiotic stress element	38
MBS	49	MYB binding site involved in drought-inducibility	Abiotic stress element	42
TC-rich repeats	27	cis-acting element involved in defense and stress responsiveness	Abiotic stress element	25
MBSI	5	MYB binding site involved in flavonoid biosynthetic genes regulation	Abiotic stress element	5
WUN-motif	46	wound-responsive element	Abiotic stress element	35
W box	151		Abiotic stress element	80
G-Box	462	cis-acting regulatory element involved in light responsiveness	Light response	100
TCCC-motif	80	part of a light responsive element	Light response	49

TCT-motif	66	part of a light responsive element	Light response	47
GATA-motif	61	part of a light responsive element	Light response	40
I-box	55	part of a light responsive element	Light response	40
GA-motif	14	part of a light responsive element	Light response	11
LAMP-element	14	part of a light responsive element	Light response	13
chs-CMA2a	13	part of a light responsive element	Light response	11
chs-CMA1a	10	part of a light responsive element	Light response	10
L-box	10	part of a light responsive element	Light response	10
Gap-box	7	part of a light responsive element	Light response	7
Box II	6	part of a light responsive element	Light response	6
GTGGC-motif	4	part of a light responsive element	Light response	4
GATT-motif	3	part of a light responsive element	Light response	3
chs-Unit 1 m1	2	part of a light responsive element	Light response	2
LS7	2	part of a light responsive element	Light response	2
chs-CMA2b	1	part of a light responsive element	Light response	1
Box 4	190	part of a conserved DNA module involved in light responsiveness	Light response	80
ATCT-motif	16	part of a conserved DNA module involved in light responsiveness	Light response	15
ATC-motif	8	part of a conserved DNA module involved in light responsiveness	Light response	8
GT1-motif	99	light responsive element	Light response	57
Sp1	61	light responsive element	Light response	34
3-AF1 binding site	12	light responsive element	Light response	10
AAAC-motif	1	light responsive element	Light response	1
MRE	33	MYB binding site involved in light responsiveness	Light response	27
AE-box	32	part of a module for light response	Light response	28
ACE	21	cis-acting element involved in light responsiveness	Light response	19
CAG-motif	7	part of a light response element	Light response	7
AT1-motif	4	part of a light responsive module	Light response	4
HD-Zip 1	1	element involved in differentiation of the palisade mesophyll cells	Light response	1
O2-site	70	cis-acting regulatory element involved in zein metabolism regulation	Growth and development element	54
A-box	59	cis-acting regulatory element	Growth and development element	39
CAT-box	44	cis-acting regulatory element related to meristem expression	Growth and development element	36
GCN4_motif	27	cis-regulatory element involved in endosperm expressionCis	Growth and development element	23

RY-element	9	cis-acting regulatory element involved in seed-specific regulation	Growth and development element	9
MSA-like	1	cis-acting element involved in cell cycle regulation	Growth and development element	1

Table S4. Ka/Ks analysis of *EgWRKY* genes in *E. grandis*.

Seq 1	Seq 2	Ka	Ks	Ka/Ks	Duplication type	Purify selection
EgWRKY86	EgWRKY88	0.1509883799698052	0.44920767208550527	0.3361215521293791	Tandem	Yes
EgWRKY1	EgWRKY5	0.15058819079952107	0.4654598892346329	0.3235256018453855	Segmental	Yes
EgWRKY8	EgWRKY111	0.2693324070228317	1.2361539102411623	0.2178793472167939	Segmental	Yes
EgWRKY9	EgWRKY111	0.2693324070228317	1.2361539102411623	0.2178793472167939	Segmental	Yes
EgWRKY11	EgWRKY40	0.47047657566122447	NaN	NaN	Segmental	/
EgWRKY13	EgWRKY59	0.5298641475176831	2.3296663450857245	0.2274420749715495	Segmental	Yes
EgWRKY15	EgWRKY106	0.3522696293251416	2.2589051173000243	0.15594706773084605	Segmental	Yes
EgWRKY16	EgWRKY36	0.4731753184497581	1.8630353805114521	0.25398085479184995	Segmental	Yes
EgWRKY16	EgWRKY37	0.47411862531850174	1.2487055225675854	0.37968809839458395	Segmental	Yes
EgWRKY18	EgWRKY66	0.27011968252960766	2.1184945719719437	0.12750548719989122	Segmental	Yes
EgWRKY18	EgWRKY75	0.4737091083316113	3.859485082475781	0.1227389400939818	Segmental	Yes
EgWRKY21	EgWRKY73	0.33330080412172924	2.0079170529802113	0.16599331313364468	Segmental	Yes
EgWRKY22	EgWRKY74	0.26762255015547687	1.309215822428095	0.20441438727736985	Segmental	Yes
EgWRKY35	EgWRKY38	0.39550698246061644	1.7897938307001442	0.2209790734980348	Segmental	Yes
EgWRKY36	EgWRKY37	0.542416993254972	1.8247021445146667	0.29726330671861173	Segmental	Yes
EgWRKY38	EgWRKY40	0.4034611295397792	2.451606145212315	0.16457012490676332	Segmental	Yes
EgWRKY37	EgWRKY41	0.35458871699642247	1.1993145724999679	0.2956594751094229	Segmental	Yes
EgWRKY37	EgWRKY42	0.35594677081525106	1.191232774506163	0.298805387522025	Segmental	Yes
EgWRKY51	EgWRKY59	0.2710223800861029	1.0012905309387112	0.2706730681174215	Segmental	Yes
EgWRKY60	EgWRKY101	0.23673099869960057	1.1571879225694013	0.20457437731804778	Segmental	Yes

EgWRKY75	EgWRKY93	0.48649808747649403	NaN	NaN	Segmental	/
EgWRKY76	EgWRKY98	0.3785408447931545	1.9300730421796102	0.19612773015350365	Segmental	Yes
EgWRKY77	EgWRKY96	0.41972765989844757	1.5730433390853982	0.26682523581485457	Segmental	Yes
EgWRKY78	EgWRKY80	0.47876992605892055	1.8160670461800295	0.2636300939802744	Segmental	Yes
EgWRKY103	EgWRKY108	0.3212393871628488	2.4886546648425054	0.12908154421785895	Segmental	Yes

Table S5. Genetic linkage pairs between *E. grandis* and five other plant species.

Gene Name	Seq1 Gene ID	Chromosome Position	Species name	Seq2 GeneID	Chromosome Position
EgWRKY9	rna-XM_010048642.3	Chr01	*Arabidopsis thaliana*	AT2G37260	2
EgWRKY12	rna-XM_010047269.3	Chr01	*Arabidopsis thaliana*	AT4G01720	4
EgWRKY13	rna-XM_010047652.3	Chr01	*Arabidopsis thaliana*	AT4G26640	4
EgWRKY13	rna-XM_010047652.3	Chr01	*Arabidopsis thaliana*	AT2G04880	2
EgWRKY16	rna-XM_010061956.3	Chr01	*Arabidopsis thaliana*	AT5G52830	5
EgWRKY17	rna-XM_010048806.3	Che01	*Arabidopsis thaliana*	AT4G30935	4
EgWRKY18	rna-XM_010030811.3	Chr02	*Arabidopsis thaliana*	AT1G18860	1
EgWRKY21	rna-XM_010048316.3	Chr02	*Arabidopsis thaliana*	AT1G69310	1
EgWRKY22	rna-XM_010046160.3	Chr02	*Arabidopsis thaliana*	AT1G13960	1
EgWRKY22	rna-XM_010046160.3	Chr02	*Arabidopsis thaliana*	AT2G03340	2
EgWRKY23	rna-XM_010046542.3	Chr02	*Arabidopsis thaliana*	AT5G13080	5
EgWRKY24	rna-XM_010047093.3	Chr02	*Arabidopsis thaliana*	AT2G30250	2
EgWRKY25	rna-XM_010049069.3	Chr03	*Arabidopsis thaliana*	AT5G26170	5
EgWRKY33	rna-XM_010051525.3	Chr03	*Arabidopsis thaliana*	AT2G24570	2
EgWRKY33	rna-XM_010051525.3	Chr03	*Arabidopsis thaliana*	AT4G31550	4
EgWRKY34	rna-XM_010053801.3	Chr04	*Arabidopsis thaliana*	AT4G18170	4
EgWRKY34	rna-XM_010053801.3	Chr04	*Arabidopsis thaliana*	AT5G46350	5
EgWRKY34	rna-XM_010053801.3	Chr04	*Arabidopsis thaliana*	AT1G29860	1
EgWRKY35	rna-XM_010054903.3	Chr04	*Arabidopsis thaliana*	AT2G46130	2
EgWRKY35	rna-XM_010054903.3	Chr04	*Arabidopsis thaliana*	AT5G41570	5
EgWRKY35	rna-XM_010054903.3	Chr04	*Arabidopsis thaliana*	AT1G64000	1
EgWRKY36	rna-XM_010054907.3	Chr04	*Arabidopsis thaliana*	AT4G23550	4
EgWRKY36	rna-XM_010054907.3	Chr04	*Arabidopsis thaliana*	AT5G52830	5
EgWRKY37	rna-XM_010055460.3	Chr04	*Arabidopsis thaliana*	AT4G01250	4
EgWRKY38	rna-XM_010055462.3	Chr04	*Arabidopsis thaliana*	AT1G64000	1
EgWRKY38	rna-XM_010055462.3	Chr04	*Arabidopsis thaliana*	AT2G46130	2
EgWRKY39	rna-XM_010055498.3	Chr04	*Arabidopsis thaliana*	AT2G46400	2
EgWRKY40	rna-XM_010056773.3	Chr05	*Arabidopsis thaliana*	AT2G46130	2
EgWRKY41	rna-XM_010056776.3	Chr05	*Arabidopsis thaliana*	AT4G01250	4
EgWRKY43	rna-XM_010054488.3	Chr05	*Arabidopsis thaliana*	AT2G44745	2
EgWRKY47	rna-XM_010038330.3	Chr05	*Arabidopsis thaliana*	AT1G30650	1
EgWRKY50	rna-XM_010061595.3	Chr06	*Arabidopsis thaliana*	AT2G23320	2
EgWRKY51	rna-XM_010061938.3	Chr06	*Arabidopsis thaliana*	AT4G26640	4
EgWRKY55	rna-XM_039315841.1	Chr06	*Arabidopsis thaliana*	AT5G56270	5
EgWRKY55	rna-XM_039315841.1	Chr06	*Arabidopsis thaliana*	AT4G26440	4
EgWRKY59	rna-XM_010064779.3	Chr06	*Arabidopsis thaliana*	AT4G26640	4
EgWRKY60	rna-XM_039315439.1	Chr06	*Arabidopsis thaliana*	AT1G80840	1
EgWRKY73	rna-XM_010068554.3	Chr07	*Arabidopsis thaliana*	AT1G69310	1
EgWRKY74	rna-XM_010068568.3	Chr07	*Arabidopsis thaliana*	AT1G13960	1
EgWRKY74	rna-XM_010068568.3	Chr07	*Arabidopsis thaliana*	AT2G03340	2

EgWRKY75	rna-XM_010068644.3	Chr07	*Arabidopsis thaliana*	AT5G15130	5
EgWRKY75	rna-XM_010068644.3	Chr07	*Arabidopsis thaliana*	AT1G69810	1
EgWRKY76	rna-XM_010069318.3	Chr07	*Arabidopsis thaliana*	AT2G40740	2
EgWRKY77	rna-XM_010069319.3	Chr07	*Arabidopsis thaliana*	AT2G40750	2
EgWRKY78	rna-XM_010071522.3	Chr08	*Arabidopsis thaliana*	AT2G47260	2
EgWRKY78	rna-XM_010071522.3	Chr08	*Arabidopsis thaliana*	AT3G62340	3
EgWRKY78	rna-XM_010071522.3	Chr08	*Arabidopsis thaliana*	AT5G49520	5
EgWRKY80	rna-XM_010026505.3	Chr08	*Arabidopsis thaliana*	AT5G49520	5
EgWRKY85	rna-XM_010029452.3	Chr09	*Arabidopsis thaliana*	AT4G39410	4
EgWRKY86	rna-XM_010029460.3	Chr09	*Arabidopsis thaliana*	AT5G64810	5
EgWRKY86	rna-XM_010029460.3	Chr09	*Arabidopsis thaliana*	AT2G21900	2
EgWRKY92	rna-XM_010030682.3	Chr09	*Arabidopsis thaliana*	AT3G01970	3
EgWRKY93	rna-XM_010030989.3	Chr09	*Arabidopsis thaliana*	AT5G15130	5
EgWRKY93	rna-XM_010030989.3	Chr09	*Arabidopsis thaliana*	AT1G69810	1
EgWRKY94	rna-XM_010031058.3	Chr09	*Arabidopsis thaliana*	AT3G01080	3
EgWRKY96	rna-XM_039303323.1	Chr10	*Arabidopsis thaliana*	AT3G56400	3
EgWRKY99	rna-XM_039303157.1	Chr10	*Arabidopsis thaliana*	AT2G40740	2
EgWRKY100	rna-XM_010057549.3	Chr10	*Arabidopsis thaliana*	AT4G11070	4
EgWRKY100	rna-XM_010057549.3	Chr10	*Arabidopsis thaliana*	AT4G23810	4
EgWRKY101	rna-XM_010035731.3	Chr10	*Arabidopsis thaliana*	AT1G80840	1
EgWRKY102	rna-XM_010036918.3	Chr11	*Arabidopsis thaliana*	AT2G46400	2
EgWRKY102	rna-XM_010036918.3	Chr11	*Arabidopsis thaliana*	AT5G24110	5
EgWRKY103	rna-XM_010037251.3	Chr11	*Arabidopsis thaliana*	AT1G62300	1
EgWRKY103	rna-XM_010037251.3	Chr11	*Arabidopsis thaliana*	AT4G04450	4
EgWRKY104	rna-XM_010037575.3	Chr11	*Arabidopsis thaliana*	AT3G58710	3
EgWRKY108	rna-XM_010059605.3	Chr11	*Arabidopsis thaliana*	AT4G04450	4
EgWRKY108	rna-XM_010059605.3	Chr11	*Arabidopsis thaliana*	AT4G22070	4
EgWRKY108	rna-XM_010059605.3	Chr11	*Arabidopsis thaliana*	AT1G62300	1
EgWRKY110	rna-XM_010039355.3	Chr11	*Arabidopsis thaliana*	AT2G38470	2
EgWRKY111	rna-XM_010039605.3	Chr11	*Arabidopsis thaliana*	AT2G37260	2
EgWRKY4	rna-XM_039316933.1	Chr01	*Populus trichocarpa*	rna-XM_002309150.4	6
EgWRKY4	rna-XM_039316933.1	Chr01	*Populus trichocarpa*	rna-XM_002323639.4	16
EgWRKY9	rna-XM_010048642.3	Chr01	*Populus trichocarpa*	rna-XM_024587515.2	16
EgWRKY12	rna-XM_010047269.3	Chr01	*Populus trichocarpa*	rna-XM_002302772.4	2
EgWRKY12	rna-XM_010047269.3	Chr01	*Populus trichocarpa*	rna-XM_024584301.2	14
EgWRKY13	rna-XM_010047652.3	Chr01	*Populus trichocarpa*	rna-XM_024584519.2	14
EgWRKY14	rna-XM_010048600.3	Chr01	*Populus trichocarpa*	rna-XM_002325212.4	18
EgWRKY14	rna-XM_010048600.3	Chr01	*Populus trichocarpa*	rna-XM_002308962.4	6
EgWRKY15	rna-XM_010055259.3	Chr01	*Populus trichocarpa*	rna-XM_002302104.4	2
EgWRKY15	rna-XM_010055259.3	Chr01	*Populus trichocarpa*	rna-XM_002306707.4	5
EgWRKY16	rna-XM_010061956.3	Chr01	*Populus trichocarpa*	rna-XM_006375289.3	14
EgWRKY16	rna-XM_010061956.3	Chr01	*Populus trichocarpa*	rna-XM_006372308.3	17
EgWRKY16	rna-XM_010061956.3	Chr01	*Populus trichocarpa*	rna-XM_006368706.3	1
EgWRKY16	rna-XM_010061956.3	Chr01	*Populus trichocarpa*	rna-XM_006386554.3	2

EgWRKY16	rna-XM_010061956.3	Chr01	*Populus trichocarpa*	rna-XM_002303574.4	3
EgWRKY16	rna-XM_010061956.3	Chr01	*Populus trichocarpa*	rna-XM_002305235.4	4
EgWRKY17	rna-XM_010048806.3	Chr01	*Populus trichocarpa*	rna-XM_024590285.2	18
EgWRKY17	rna-XM_010048806.3	Chr01	*Populus trichocarpa*	rna-XM_024604661.2	6
EgWRKY18	rna-XM_010030811.3	Chr02	*Populus trichocarpa*	rna-XM_052447365.1	15
EgWRKY18	rna-XM_010030811.3	Chr02	*Populus trichocarpa*	rna-XM_002323803.4	17
EgWRKY19	rna-XM_010040631.3	Chr02	*Populus trichocarpa*	rna-XM_002302034.4	2
EgWRKY19	rna-XM_010040631.3	Chr02	*Populus trichocarpa*	rna-XM_002306787.4	5
EgWRKY19	rna-XM_010040631.3	Chr02	*Populus trichocarpa*	rna-XM_006382750.3	5
EgWRKY19	rna-XM_010040631.3	Chr02	*Populus trichocarpa*	rna-XM_002319046.4	13
EgWRKY20	rna-XM_039304060.1	Chr02	*Populus trichocarpa*	rna-XM_024583061.2	13
EgWRKY21	rna-XM_010048316.3	Chr02	*Populus trichocarpa*	rna-XM_024606155.2	8
EgWRKY21	rna-XM_010048316.3	Chr02	*Populus trichocarpa*	rna-XM_024611133.2	10
EgWRKY22	rna-XM_010046160.3	Chr02	*Populus trichocarpa*	rna-XM_002314988.4	10
EgWRKY22	rna-XM_010046160.3	Chr02	*Populus trichocarpa*	rna-XM_024588647.2	17
EgWRKY22	rna-XM_010046160.3	Chr02	*Populus trichocarpa*	rna-XM_002312231.4	8
EgWRKY23	rna-XM_010046542.3	Chr02	*Populus trichocarpa*	rna-XM_002318711.4	12
EgWRKY23	rna-XM_010046542.3	Chr02	*Populus trichocarpa*	rna-XM_002322238.4	15
EgWRKY23	rna-XM_010046542.3	Chr02	*Populus trichocarpa*	rna-XM_006368806.3	1
EgWRKY23	rna-XM_010046542.3	Chr02	*Populus trichocarpa*	rna-XM_002300084.4	1
EgWRKY23	rna-XM_010046542.3	Chr02	*Populus trichocarpa*	rna-XM_002304705.4	3
EgWRKY24	rna-XM_010047093.3	Chr02	*Populus trichocarpa*	rna-XM_002319923.4	13
EgWRKY25	rna-XM_010049069.3	Chr03	*Populus trichocarpa*	rna-XM_002308502.3	6
EgWRKY26	rna-XM_010052520.3	Chr03	*Populus trichocarpa*	rna-XM_002324292.4	18
EgWRKY26	rna-XM_010052520.3	Chr03	*Populus trichocarpa*	rna-XM_002308668.3	6
EgWRKY27	rna-XM_010050501.3	Chr03	*Populus trichocarpa*	rna-XM_024590285.2	18
EgWRKY27	rna-XM_010050501.3	Chr03	*Populus trichocarpa*	rna-XM_024604661.2	6
EgWRKY31	rna-XM_010052826.3	Chr03	*Populus trichocarpa*	rna-XM_002308668.3	6
EgWRKY33	rna-XM_010051525.3	Chr03	*Populus trichocarpa*	rna-XM_002324346.4	18
EgWRKY34	rna-XM_010053801.3	Chr04	*Populus trichocarpa*	rna-XM_006369831.3	1
EgWRKY34	rna-XM_010053801.3	Chr04	*Populus trichocarpa*	rna-XM_024581109.2	11
EgWRKY35	rna-XM_010054903.3	Chr04	*Populus trichocarpa*	rna-XM_002320124.4	14
EgWRKY35	rna-XM_010054903.3	Chr04	*Populus trichocarpa*	rna-XM_002301341.4	2
EgWRKY36	rna-XM_010054907.3	Chr04	*Populus trichocarpa*	rna-XM_006386554.3	2
EgWRKY36	rna-XM_010054907.3	Chr04	*Populus trichocarpa*	rna-XM_002303574.4	3
EgWRKY36	rna-XM_010054907.3	Chr04	*Populus trichocarpa*	rna-XM_002305235.4	4
EgWRKY36	rna-XM_010054907.3	Chr04	*Populus trichocarpa*	rna-XM_006375289.3	14
EgWRKY36	rna-XM_010054907.3	Chr04	*Populus trichocarpa*	rna-XM_006372308.3	17
EgWRKY36	rna-XM_010054907.3	Chr04	*Populus trichocarpa*	rna-XM_006368706.3	1
EgWRKY37	rna-XM_010055460.3	Chr04	*Populus trichocarpa*	rna-XM_006368706.3	1
EgWRKY37	rna-XM_010055460.3	Chr04	*Populus trichocarpa*	rna-XM_006386554.3	2
EgWRKY37	rna-XM_010055460.3	Chr04	*Populus trichocarpa*	rna-XM_002303574.4	3
EgWRKY37	rna-XM_010055460.3	Chr04	*Populus trichocarpa*	rna-XM_006375289.3	14
EgWRKY38	rna-XM_010055462.3	Chr04	*Populus trichocarpa*	rna-XM_002320124.4	14

EgWRKY38	rna-XM_010055462.3	Chr04	*Populus trichocarpa*	rna-XM_002301341.4	2
EgWRKY39	rna-XM_010055498.3	Chr04	*Populus trichocarpa*	rna-XM_002302584.4	2
EgWRKY39	rna-XM_010055498.3	Chr04	*Populus trichocarpa*	rna-XM_002318337.4	11
EgWRKY39	rna-XM_010055498.3	Chr04	*Populus trichocarpa*	rna-XM_002320816.4	14
EgWRKY40	rna-XM_010056773.3	Chr05	*Populus trichocarpa*	rna-XM_002301341.4	2
EgWRKY40	rna-XM_010056773.3	Chr05	*Populus trichocarpa*	rna-XM_002320124.4	14
EgWRKY41	rna-XM_010056776.3	Chr05	*Populus trichocarpa*	rna-XM_006375289.3	14
EgWRKY41	rna-XM_010056776.3	Chr05	*Populus trichocarpa*	rna-XM_006368706.3	1
EgWRKY41	rna-XM_010056776.3	Chr05	*Populus trichocarpa*	rna-XM_006386554.3	2
EgWRKY43	rna-XM_010054488.3	Chr05	*Populus trichocarpa*	rna-XM_006375106.3	14
EgWRKY43	rna-XM_010054488.3	Chr05	*Populus trichocarpa*	rna-XM_002301201.4	2
EgWRKY47	rna-XM_010038330.3	Chr05	*Populus trichocarpa*	rna-XM_052445140.1	11
EgWRKY47	rna-XM_010038330.3	Chr05	*Populus trichocarpa*	rna-XM_024602795.2	1
EgWRKY48	rna-XM_018872938.2	Chr05	*Populus trichocarpa*	rna-XM_002305694.4	4
EgWRKY48	rna-XM_018872938.2	Chr05	*Populus trichocarpa*	rna-XM_002317361.4	11
EgWRKY50	rna-XM_010061595.3	Chr06	*Populus trichocarpa*	rna-XM_002301130.4	2
EgWRKY50	rna-XM_010061595.3	Chr06	*Populus trichocarpa*	rna-XM_024600511.2	5
EgWRKY50	rna-XM_010061595.3	Chr06	*Populus trichocarpa*	rna-XM_002310086.4	7
EgWRKY50	rna-XM_010061595.3	Chr06	*Populus trichocarpa*	rna-XM_024585549.2	14
EgWRKY51	rna-XM_010061938.3	Chr06	*Populus trichocarpa*	rna-XM_002298817.4	1
EgWRKY51	rna-XM_010061938.3	Chr06	*Populus trichocarpa*	rna-XM_024581601.2	11
EgWRKY55	rna-XM_039315841.1	Chr06	*Populus trichocarpa*	rna-XM_006377952.3	11
EgWRKY55	rna-XM_039315841.1	Chr06	*Populus trichocarpa*	rna-XM_024585445.2	1
EgWRKY57	rna-XM_010039521.3	Chr06	*Populus trichocarpa*	rna-XM_002309030.4	6
EgWRKY59	rna-XM_010064779.3	Chr06	*Populus trichocarpa*	rna-XM_024581601.2	11
EgWRKY59	rna-XM_010064779.3	Chr06	*Populus trichocarpa*	rna-XM_002298817.4	1
EgWRKY60	rna-XM_039315439.1	Chr06	*Populus trichocarpa*	rna-XM_024610899.2	1
EgWRKY60	rna-XM_039315439.1	Chr06	*Populus trichocarpa*	rna-XM_006368449.3	1
EgWRKY60	rna-XM_039315439.1	Chr06	*Populus trichocarpa*	rna-XM_002303816.4	3
EgWRKY60	rna-XM_039315439.1	Chr06	*Populus trichocarpa*	rna-XM_002308668.3	6
EgWRKY60	rna-XM_039315439.1	Chr06	*Populus trichocarpa*	rna-XM_002324292.4	18
EgWRKY66	rna-XM_010038852.3	Chr07	*Populus trichocarpa*	rna-XM_052447365.1	15
EgWRKY66	rna-XM_010038852.3	Chr07	*Populus trichocarpa*	rna-XM_002323803.4	17
EgWRKY73	rna-XM_010068554.3	Chr07	*Populus trichocarpa*	rna-XM_024606155.2	8
EgWRKY73	rna-XM_010068554.3	Chr07	*Populus trichocarpa*	rna-XM_024611133.2	10
EgWRKY74	rna-XM_010068568.3	Chr07	*Populus trichocarpa*	rna-XM_002314988.4	10
EgWRKY74	rna-XM_010068568.3	Chr07	*Populus trichocarpa*	rna-XM_024588647.2	17
EgWRKY74	rna-XM_010068568.3	Chr07	*Populus trichocarpa*	rna-XM_002312231.4	8
EgWRKY75	rna-XM_010068644.3	Chr07	*Populus trichocarpa*	rna-XM_052447365.1	15
EgWRKY75	rna-XM_010068644.3	Chr07	*Populus trichocarpa*	rna-XM_002323803.4	17
EgWRKY76	rna-XM_010069318.3	Chr07	*Populus trichocarpa*	rna-XM_052449326.1	19
EgWRKY76	rna-XM_010069318.3	Chr07	*Populus trichocarpa*	rna-XM_002309150.4	6
EgWRKY76	rna-XM_010069318.3	Chr07	*Populus trichocarpa*	rna-XM_024583529.2	13
EgWRKY76	rna-XM_010069318.3	Chr07	*Populus trichocarpa*	rna-XM_002323639.4	16

EgWRKY77	rna-XM_010069319.3	Chr07	*Populus trichocarpa*	rna-XM_052446276.1	13
EgWRKY78	rna-XM_010071522.3	Chr08	*Populus trichocarpa*	rna-XM_002301488.4	2
EgWRKY78	rna-XM_010071522.3	Chr08	*Populus trichocarpa*	rna-XM_002320931.4	14
EgWRKY79	rna-XM_010071532.3	Chr08	*Populus trichocarpa*	rna-XM_024596020.2	2
EgWRKY79	rna-XM_010071532.3	Chr08	*Populus trichocarpa*	rna-XM_052447277.1	14
EgWRKY80	rna-XM_010026505.3	Chr08	*Populus trichocarpa*	rna-XM_002301488.4	2
EgWRKY80	rna-XM_010026505.3	Chr08	*Populus trichocarpa*	rna-XM_002312282.4	8
EgWRKY80	rna-XM_010026505.3	Chr08	*Populus trichocarpa*	rna-XM_002314926.4	10
EgWRKY80	rna-XM_010026505.3	Chr08	*Populus trichocarpa*	rna-XM_002320931.4	14
EgWRKY85	rna-XM_010029452.3	Chr09	*Populus trichocarpa*	rna-XM_052452688.1	5
EgWRKY85	rna-XM_010029452.3	Chr09	*Populus trichocarpa*	rna-XM_002310008.4	7
EgWRKY86	rna-XM_010029460.3	Chr09	*Populus trichocarpa*	rna-XM_024605234.2	7
EgWRKY86	rna-XM_010029460.3	Chr09	*Populus trichocarpa*	rna-XM_024601099.2	5
EgWRKY91	rna-XM_039302236.1	Chr09	*Populus trichocarpa*	rna-XM_024600511.2	5
EgWRKY91	rna-XM_039302236.1	Chr09	*Populus trichocarpa*	rna-XM_002310086.4	7
EgWRKY91	rna-XM_039302236.1	Chr09	*Populus trichocarpa*	rna-XM_024585549.2	14
EgWRKY91	rna-XM_039302236.1	Chr09	*Populus trichocarpa*	rna-XM_002301130.4	2
EgWRKY92	rna-XM_010030682.3	Chr09	*Populus trichocarpa*	rna-XM_002318711.4	12
EgWRKY92	rna-XM_010030682.3	Chr09	*Populus trichocarpa*	rna-XM_002300084.4	1
EgWRKY93	rna-XM_010030989.3	Chr09	*Populus trichocarpa*	rna-XM_052447365.1	15
EgWRKY93	rna-XM_010030989.3	Chr09	*Populus trichocarpa*	rna-XM_002323803.4	17
EgWRKY94	rna-XM_010031058.3	Chr09	*Populus trichocarpa*	rna-XM_002314988.4	10
EgWRKY94	rna-XM_010031058.3	Chr09	*Populus trichocarpa*	rna-XM_024588647.2	17
EgWRKY94	rna-XM_010031058.3	Chr09	*Populus trichocarpa*	rna-XM_024598510.2	4
EgWRKY96	rna-XM_039303323.1	Chr10	*Populus trichocarpa*	rna-XM_052446276.1	12
EgWRKY96	rna-XM_039303323.1	Chr10	*Populus trichocarpa*	rna-XM_002323639.4	16
EgWRKY99	rna-XM_039303157.1	Chr10	*Populus trichocarpa*	rna-XM_052449326.1	19
EgWRKY99	rna-XM_039303157.1	Chr10	*Populus trichocarpa*	rna-XM_024583529.2	13
EgWRKY100	rna-XM_010057549.3	Chr10	*Populus trichocarpa*	rna-XM_002297947.4	1
EgWRKY100	rna-XM_010057549.3	Chr10	*Populus trichocarpa*	rna-XM_002304513.4	3
EgWRKY101	rna-XM_010035731.3	Chr10	*Populus trichocarpa*	rna-XM_002308668.3	6
EgWRKY101	rna-XM_010035731.3	Chr10	*Populus trichocarpa*	rna-XM_002324292.4	18
EgWRKY101	rna-XM_010035731.3	Chr10	*Populus trichocarpa*	rna-XM_006368449.3	1
EgWRKY101	rna-XM_010035731.3	Chr10	*Populus trichocarpa*	rna-XM_002303816.4	3
EgWRKY102	rna-XM_010036918.3	Chr11	*Populus trichocarpa*	rna-XM_002302584.4	2
EgWRKY102	rna-XM_010036918.3	Chr11	*Populus trichocarpa*	rna-XM_002320816.4	14
EgWRKY103	rna-XM_010037251.3	Chr11	*Populus trichocarpa*	rna-XM_002321098.4	14
EgWRKY103	rna-XM_010037251.3	Chr11	*Populus trichocarpa*	rna-XM_024600078.2	4
EgWRKY103	rna-XM_010037251.3	Chr11	*Populus trichocarpa*	rna-XM_024611531.2	11
EgWRKY104	rna-XM_010037575.3	Chr11	*Populus trichocarpa*	rna-XM_002305694.4	4
EgWRKY104	rna-XM_010037575.3	Chr11	*Populus trichocarpa*	rna-XM_002317361.4	11
EgWRKY106	rna-XM_010037620.3	Chr11	*Populus trichocarpa*	rna-XM_002302104.4	2
EgWRKY106	rna-XM_010037620.3	Chr11	*Populus trichocarpa*	rna-XM_002306707.4	5
EgWRKY107	rna-XM_010037704.3	Chr11	*Populus trichocarpa*	rna-XM_002309150.4	6

EgWRKY107	rna-XM_010037704.3	Chr11	*Populus trichocarpa*	rna-XM_002323639.4	16
EgWRKY108	rna-XM_010059605.3	Chr11	*Populus trichocarpa*	rna-XM_024600078.2	4
EgWRKY108	rna-XM_010059605.3	Chr11	*Populus trichocarpa*	rna-XM_024611531.2	11
EgWRKY108	rna-XM_010059605.3	Chr11	*Populus trichocarpa*	rna-XM_002321098.4	14
EgWRKY110	rna-XM_010039355.3	Chr11	*Populus trichocarpa*	rna-XM_024602932.2	6
EgWRKY110	rna-XM_010039355.3	Chr11	*Populus trichocarpa*	rna-XM_002323601.4	16
EgWRKY111	rna-XM_010039605.3	Chr11	*Populus trichocarpa*	rna-XM_006381457.3	6
EgWRKY111	rna-XM_010039605.3	Chr11	*Populus trichocarpa*	rna-XM_024587515.2	16
EgWRKY1	rna-XM_010045152.3	Chr01	*Malus domestica*	rna-XM_008357516.4	4
EgWRKY4	rna-XM_039316933.1	Chr01	*Malus domestica*	rna-XM_070825322.1	7
EgWRKY4	rna-XM_039316933.1	Chr01	*Malus domestica*	rna-XM_070810121.1	12
EgWRKY4	rna-XM_039316933.1	Chr01	*Malus domestica*	rna-XM_008390142.3	1
EgWRKY9	rna-XM_010048642.3	Chr01	*Malus domestica*	rna-XM_008389468.4	12
EgWRKY9	rna-XM_010048642.3	Chr01	*Malus domestica*	rna-XM_008371590.4	4
EgWRKY13	rna-XM_010047652.3	Chr01	*Malus domestica*	rna-XM_008381756.4	9
EgWRKY13	rna-XM_010047652.3	Chr01	*Malus domestica*	rna-XM_029088856.2	11
EgWRKY13	rna-XM_010047652.3	Chr01	*Malus domestica*	rna-XM_029097659.2	17
EgWRKY14	rna-XM_010048600.3	Chr01	*Malus domestica*	rna-XM_008365700.4	13
EgWRKY14	rna-XM_010048600.3	Chr01	*Malus domestica*	rna-XM_029096103.2	16
EgWRKY15	rna-XM_010055259.3	Chr01	*Malus domestica*	rna-XM_008344130.4	17
EgWRKY15	rna-XM_010055259.3	Chr01	*Malus domestica*	rna-XM_008373350.4	5
EgWRKY15	rna-XM_010055259.3	Chr01	*Malus domestica*	rna-XM_070804849.1	9
EgWRKY15	rna-XM_010055259.3	Chr01	*Malus domestica*	rna-XM_008385286.4	10
EgWRKY16	rna-XM_010061956.3	Chr01	*Malus domestica*	rna-XM_008365060.4	14
EgWRKY16	rna-XM_010061956.3	Chr01	*Malus domestica*	rna-XM_008382015.4	1
EgWRKY16	rna-XM_010061956.3	Chr01	*Malus domestica*	rna-XM_029099136.2	1
EgWRKY16	rna-XM_010061956.3	Chr01	*Malus domestica*	rna-XM_008377742.4	6
EgWRKY16	rna-XM_010061956.3	Chr01	*Malus domestica*	rna-XM_008348902.4	7
EgWRKY16	rna-XM_010061956.3	Chr01	*Malus domestica*	rna-XM_017333280.3	7
EgWRKY17	rna-XM_010048806.3	Chr01	*Malus domestica*	rna-XM_008396100.4	15
EgWRKY17	rna-XM_010048806.3	Chr01	*Malus domestica*	rna-XM_008386160.4	2
EgWRKY18	rna-XM_010030811.3	Chr02	*Malus domestica*	rna-XM_008376310.4	6
EgWRKY18	rna-XM_010030811.3	Chr02	*Malus domestica*	rna-XM_029107805.2	9
EgWRKY18	rna-XM_010030811.3	Chr02	*Malus domestica*	rna-XM_070810874.1	13
EgWRKY18	rna-XM_010030811.3	Chr02	*Malus domestica*	rna-XM_029093233.2	14
EgWRKY18	rna-XM_010030811.3	Chr02	*Malus domestica*	rna-XM_070815839.1	16
EgWRKY18	rna-XM_010030811.3	Chr02	*Malus domestica*	rna-XM_008343325.4	17
EgWRKY19	rna-XM_010040631.3	Chr02	*Malus domestica*	rna-NM_001294069.1	10
EgWRKY19	rna-XM_010040631.3	Chr02	*Malus domestica*	rna-XM_029090147.2	12
EgWRKY19	rna-XM_010040631.3	Chr02	*Malus domestica*	rna-XM_008347498.4	4
EgWRKY19	rna-XM_010040631.3	Chr02	*Malus domestica*	rna-XM_029102680.2	5
EgWRKY20	rna-XM_039304060.1	Chr02	*Malus domestica*	rna-XM_029090222.2	12
EgWRKY20	rna-XM_039304060.1	Chr02	*Malus domestica*	rna-XM_017331770.3	4
EgWRKY21	rna-XM_010048316.3	Chr02	*Malus domestica*	rna-XM_070810772.1	13

EgWRKY21	rna-XM_010048316.3	Chr02	*Malus domestica*	rna-XM_029095804.2	16
EgWRKY22	rna-XM_010046160.3	Chr02	*Malus domestica*	rna-XM_008354749.4	16
EgWRKY22	rna-XM_010046160.3	Chr02	*Malus domestica*	rna-XM_008343388.4	17
EgWRKY22	rna-XM_010046160.3	Chr02	*Malus domestica*	rna-NM_001294081.1	9
EgWRKY22	rna-XM_010046160.3	Chr02	*Malus domestica*	rna-XM_008391217.4	13
EgWRKY23	rna-XM_010046542.3	Chr02	*Malus domestica*	rna-XM_070815478.1	16
EgWRKY23	rna-XM_010046542.3	Chr02	*Malus domestica*	rna-XM_008343449.4	17
EgWRKY23	rna-XM_010046542.3	Chr02	*Malus domestica*	rna-XM_008376006.4	6
EgWRKY23	rna-XM_010046542.3	Chr02	*Malus domestica*	rna-XM_008380876.4	9
EgWRKY23	rna-XM_010046542.3	Chr02	*Malus domestica*	rna-XM_008391676.4	13
EgWRKY24	rna-XM_010047093.3	Chr02	*Malus domestica*	rna-NM_001328755.1	3
EgWRKY24	rna-XM_010047093.3	Chr02	*Malus domestica*	rna-XM_008386494.4	11
EgWRKY25	rna-XM_010049069.3	Chr03	*Malus domestica*	rna-XM_029106795.2	8
EgWRKY25	rna-XM_010049069.3	Chr03	*Malus domestica*	rna-XM_008358240.4	15
EgWRKY26	rna-XM_010052520.3	Chr03	*Malus domestica*	rna-XM_008349270.4	8
EgWRKY26	rna-XM_010052520.3	Chr03	*Malus domestica*	rna-XM_017334243.3	15
EgWRKY27	rna-XM_010050501.3	Chr03	*Malus domestica*	rna-XM_008386160.4	2
EgWRKY27	rna-XM_010050501.3	Chr03	*Malus domestica*	rna-XM_008396100.4	15
EgWRKY31	rna-XM_010052826.3	Chr03	*Malus domestica*	rna-XM_017334243.3	15
EgWRKY31	rna-XM_010052826.3	Chr03	*Malus domestica*	rna-XM_008349270.4	8
EgWRKY33	rna-XM_010051525.3	Chr03	*Malus domestica*	rna-XM_008395718.4	15
EgWRKY33	rna-XM_010051525.3	Chr03	*Malus domestica*	rna-NM_001293911.1	8
EgWRKY34	rna-XM_010053801.3	Chr04	*Malus domestica*	rna-XM_008373350.4	5
EgWRKY34	rna-XM_010053801.3	Chr04	*Malus domestica*	rna-XM_008385286.4	10
EgWRKY35	rna-XM_010054903.3	Chr04	*Malus domestica*	rna-XM_008355785.4	1
EgWRKY36	rna-XM_010054907.3	Chr04	*Malus domestica*	rna-XM_029099136.2	1
EgWRKY36	rna-XM_010054907.3	Chr04	*Malus domestica*	rna-XM_008382015.4	1
EgWRKY36	rna-XM_010054907.3	Chr04	*Malus domestica*	rna-XM_008377742.4	6
EgWRKY36	rna-XM_010054907.3	Chr04	*Malus domestica*	rna-XM_008348902.4	7
EgWRKY36	rna-XM_010054907.3	Chr04	*Malus domestica*	rna-XM_017333280.3	7
EgWRKY36	rna-XM_010054907.3	Chr04	*Malus domestica*	rna-XM_008365060.4	14
EgWRKY37	rna-XM_010055460.3	Chr04	*Malus domestica*	rna-XM_029099136.2	1
EgWRKY37	rna-XM_010055460.3	Chr04	*Malus domestica*	rna-XM_008382015.4	1
EgWRKY37	rna-XM_010055460.3	Chr04	*Malus domestica*	rna-XM_008377742.4	6
EgWRKY37	rna-XM_010055460.3	Chr04	*Malus domestica*	rna-XM_008348902.4	7
EgWRKY38	rna-XM_010055462.3	Chr04	*Malus domestica*	rna-XM_008355785.4	1
EgWRKY39	rna-XM_010055498.3	Chr04	*Malus domestica*	rna-NM_001293945.1	1
EgWRKY39	rna-XM_010055498.3	Chr04	*Malus domestica*	rna-XM_008345563.4	1
EgWRKY39	rna-XM_010055498.3	Chr04	*Malus domestica*	rna-XM_008377844.4	7
EgWRKY39	rna-XM_010055498.3	Chr04	*Malus domestica*	rna-XM_029106198.2	7
EgWRKY40	rna-XM_010056773.3	Chr05	*Malus domestica*	rna-XM_008355785.4	1
EgWRKY41	rna-XM_010056776.3	Chr05	*Malus domestica*	rna-XM_029099136.2	1
EgWRKY41	rna-XM_010056776.3	Chr05	*Malus domestica*	rna-XM_008348902.4	7
EgWRKY41	rna-XM_010056776.3	Chr05	*Malus domestica*	rna-XM_017333280.3	7

EgWRKY43	rna-XM_010054488.3	Chr05	*Malus domestica*	rna-XM_029105603.2	7
EgWRKY47	rna-XM_010038330.3	Chr05	*Malus domestica*	rna-XM_029110189.2	10
EgWRKY47	rna-XM_010038330.3	Chr05	*Malus domestica*	rna-XM_008369180.4	5
EgWRKY48	rna-XM_018872938.2	Chr05	*Malus domestica*	rna-NM_001293853.1	10
EgWRKY48	rna-XM_018872938.2	Chr05	*Malus domestica*	rna-XM_008373278.4	5
EgWRKY50	rna-XM_010061595.3	Chr06	*Malus domestica*	rna-NM_001293903.1	2
EgWRKY50	rna-XM_010061595.3	Chr06	*Malus domestica*	rna-XM_008366884.4	8
EgWRKY50	rna-XM_010061595.3	Chr06	*Malus domestica*	rna-XM_008386226.4	15
EgWRKY50	rna-XM_010061595.3	Chr06	*Malus domestica*	rna-XM_008395555.4	15
EgWRKY51	rna-XM_010061938.3	Chr06	*Malus domestica*	rna-XM_008343986.4	3
EgWRKY51	rna-XM_010061938.3	Chr06	*Malus domestica*	rna-XM_029088856.2	11
EgWRKY52	rna-XM_039314963.1	Chr06	*Malus domestica*	rna-XM_008368238.4	11
EgWRKY52	rna-XM_039314963.1	Chr06	*Malus domestica*	rna-XM_029100134.2	3
EgWRKY55	rna-XM_039315841.1	Chr06	*Malus domestica*	rna-XM_008346536.4	3
EgWRKY57	rna-XM_010039521.3	Chr06	*Malus domestica*	rna-XM_017331802.3	4
EgWRKY57	rna-XM_010039521.3	Chr06	*Malus domestica*	rna-XM_029090470.2	12
EgWRKY59	rna-XM_010064779.3	Chr06	*Malus domestica*	rna-XM_008343986.4	3
EgWRKY59	rna-XM_010064779.3	Chr06	*Malus domestica*	rna-XM_029088856.2	11
EgWRKY60	rna-XM_039315439.1	Chr06	*Malus domestica*	rna-XM_008344585.4	17
EgWRKY60	rna-XM_039315439.1	Chr06	*Malus domestica*	rna-XM_008394247.4	9
EgWRKY64	rna-XM_010067206.3	Chr07	*Malus domestica*	rna-XM_008367066.4	8
EgWRKY64	rna-XM_010067206.3	Chr07	*Malus domestica*	rna-XM_070813881.1	15
EgWRKY66	rna-XM_010038852.3	Chr07	*Malus domestica*	rna-XM_008343325.4	17
EgWRKY66	rna-XM_010038852.3	Chr07	*Malus domestica*	rna-XM_008376310.4	6
EgWRKY66	rna-XM_010038852.3	Chr07	*Malus domestica*	rna-XM_029107805.2	9
EgWRKY66	rna-XM_010038852.3	Chr07	*Malus domestica*	rna-XM_029093233.2	14
EgWRKY73	rna-XM_010068554.3	Chr07	*Malus domestica*	rna-XM_029095804.2	16
EgWRKY73	rna-XM_010068554.3	Chr07	*Malus domestica*	rna-XM_070810772.1	13
EgWRKY74	rna-XM_010068568.3	Chr07	*Malus domestica*	rna-XM_008391217.4	13
EgWRKY74	rna-XM_010068568.3	Chr07	*Malus domestica*	rna-XM_008354749.4	16
EgWRKY74	rna-XM_010068568.3	Chr07	*Malus domestica*	rna-XM_008343388.4	17
EgWRKY74	rna-XM_010068568.3	Chr07	*Malus domestica*	rna-NM_001294081.1	9
EgWRKY75	rna-XM_010068644.3	Chr07	*Malus domestica*	rna-XM_029107805.2	9
EgWRKY75	rna-XM_010068644.3	Chr07	*Malus domestica*	rna-XM_070810874.1	13
EgWRKY75	rna-XM_010068644.3	Chr07	*Malus domestica*	rna-XM_029093233.2	14
EgWRKY75	rna-XM_010068644.3	Chr07	*Malus domestica*	rna-XM_070815839.1	16
EgWRKY75	rna-XM_010068644.3	Chr07	*Malus domestica*	rna-XM_008343325.4	17
EgWRKY75	rna-XM_010068644.3	Chr07	*Malus domestica*	rna-XM_008376310.4	6
EgWRKY76	rna-XM_010069318.3	Chr07	*Malus domestica*	rna-XM_008371194.4	7
EgWRKY77	rna-XM_010069319.3	Chr07	*Malus domestica*	rna-XM_070825322.1	7
EgWRKY77	rna-XM_010069319.3	Chr07	*Malus domestica*	rna-XM_008390138.4	12
EgWRKY77	rna-XM_010069319.3	Chr07	*Malus domestica*	rna-XM_008390142.3	1
EgWRKY78	rna-XM_010071522.3	Chr08	*Malus domestica*	rna-XM_008383213.4	9
EgWRKY78	rna-XM_010071522.3	Chr08	*Malus domestica*	rna-XM_008344952.4	17

EgWRKY79	rna-XM_010071532.3	Chr08	*Malus domestica*	rna-XM_029100444.2	3
EgWRKY79	rna-XM_010071532.3	Chr08	*Malus domestica*	rna-NM_001293967.1	11
EgWRKY80	rna-XM_010026505.3	Chr08	*Malus domestica*	rna-NM_001294135.1	13
EgWRKY80	rna-XM_010026505.3	Chr08	*Malus domestica*	rna-XM_008342366.4	16
EgWRKY85	rna-XM_010029452.3	Chr09	*Malus domestica*	rna-NM_001328895.1	1
EgWRKY85	rna-XM_010029452.3	Chr09	*Malus domestica*	rna-XM_008393795.4	15
EgWRKY86	rna-XM_010029460.3	Chr09	*Malus domestica*	rna-XM_008344613.4	15
EgWRKY91	rna-XM_039302236.1	Chr09	*Malus domestica*	rna-XM_008386226.4	15
EgWRKY91	rna-XM_039302236.1	Chr09	*Malus domestica*	rna-NM_001293903.1	2
EgWRKY91	rna-XM_039302236.1	Chr09	*Malus domestica*	rna-XM_008366884.4	8
EgWRKY92	rna-XM_010030682.3	Chr09	*Malus domestica*	rna-XM_008380876.4	9
EgWRKY92	rna-XM_010030682.3	Chr09	*Malus domestica*	rna-XM_008391676.4	13
EgWRKY92	rna-XM_010030682.3	Chr09	*Malus domestica*	rna-XM_008343449.4	17
EgWRKY93	rna-XM_010030989.3	Chr09	*Malus domestica*	rna-XM_008376310.4	6
EgWRKY93	rna-XM_010030989.3	Chr09	*Malus domestica*	rna-XM_029107805.2	9
EgWRKY93	rna-XM_010030989.3	Chr09	*Malus domestica*	rna-XM_070810874.1	13
EgWRKY93	rna-XM_010030989.3	Chr09	*Malus domestica*	rna-XM_029093233.2	14
EgWRKY93	rna-XM_010030989.3	Chr09	*Malus domestica*	rna-XM_070815839.1	16
EgWRKY93	rna-XM_010030989.3	Chr09	*Malus domestica*	rna-XM_008343325.4	17
EgWRKY94	rna-XM_010031058.3	Chr09	*Malus domestica*	rna-NM_001294081.1	9
EgWRKY94	rna-XM_010031058.3	Chr09	*Malus domestica*	rna-XM_008391217.4	13
EgWRKY94	rna-XM_010031058.3	Chr09	*Malus domestica*	rna-XM_008354749.4	16
EgWRKY94	rna-XM_010031058.3	Chr09	*Malus domestica*	rna-XM_008343388.4	17
EgWRKY96	rna-XM_039303323.1	Chr10	*Malus domestica*	rna-XM_008390142.3	1
EgWRKY96	rna-XM_039303323.1	Chr10	*Malus domestica*	rna-XM_070825322.1	7
EgWRKY96	rna-XM_039303323.1	Chr10	*Malus domestica*	rna-XM_008390138.4	12
EgWRKY99	rna-XM_039303157.1	Chr10	*Malus domestica*	rna-XM_008371194.4	7
EgWRKY100	rna-XM_010057549.3	Chr10	*Malus domestica*	rna-XM_008377844.4	7
EgWRKY100	rna-XM_010057549.3	Chr10	*Malus domestica*	rna-XM_029092105.2	14
EgWRKY100	rna-XM_010057549.3	Chr10	*Malus domestica*	rna-XM_029103698.2	6
EgWRKY101	rna-XM_010035731.3	Chr10	*Malus domestica*	rna-XM_008394247.4	9
EgWRKY101	rna-XM_010035731.3	Chr10	*Malus domestica*	rna-XM_008344585.4	17
EgWRKY102	rna-XM_010036918.3	Chr11	*Malus domestica*	rna-XM_008345563.4	1
EgWRKY102	rna-XM_010036918.3	Chr11	*Malus domestica*	rna-NM_001293945.1	1
EgWRKY102	rna-XM_010036918.3	Chr11	*Malus domestica*	rna-XM_029106198.2	7
EgWRKY103	rna-XM_010037251.3	Chr11	*Malus domestica*	rna-XM_017334154.3	9
EgWRKY103	rna-XM_010037251.3	Chr11	*Malus domestica*	rna-XM_029087622.2	10
EgWRKY103	rna-XM_010037251.3	Chr11	*Malus domestica*	rna-XM_008368238.4	11
EgWRKY103	rna-XM_010037251.3	Chr11	*Malus domestica*	rna-XM_008343806.4	17
EgWRKY103	rna-XM_010037251.3	Chr11	*Malus domestica*	rna-XM_029100134.2	3
EgWRKY103	rna-XM_010037251.3	Chr11	*Malus domestica*	rna-XM_008365867.4	5
EgWRKY104	rna-XM_010037575.3	Chr11	*Malus domestica*	rna-XM_008373278.4	5
EgWRKY104	rna-XM_010037575.3	Chr11	*Malus domestica*	rna-XM_008382969.4	9
EgWRKY104	rna-XM_010037575.3	Chr11	*Malus domestica*	rna-NM_001293853.1	10

EgWRKY106	rna-XM_010037620.3	Chr11	*Malus domestica*	rna-XM_008344130.4	17
EgWRKY106	rna-XM_010037620.3	Chr11	*Malus domestica*	rna-XM_008373350.4	5
EgWRKY106	rna-XM_010037620.3	Chr11	*Malus domestica*	rna-XM_070804849.1	9
EgWRKY107	rna-XM_010037704.3	Chr11	*Malus domestica*	rna-XM_008390138.4	12
EgWRKY107	rna-XM_010037704.3	Chr11	*Malus domestica*	rna-XM_070820722.1	4
EgWRKY107	rna-XM_010037704.3	Chr11	*Malus domestica*	rna-XM_070825322.1	7
EgWRKY108	rna-XM_010059605.3	Chr11	*Malus domestica*	rna-XM_017334154.3	9
EgWRKY108	rna-XM_010059605.3	Chr11	*Malus domestica*	rna-XM_029087622.2	10
EgWRKY108	rna-XM_010059605.3	Chr11	*Malus domestica*	rna-XM_008365867.4	5
EgWRKY110	rna-XM_010039355.3	Chr11	*Malus domestica*	rna-XM_070809708.1	12
EgWRKY110	rna-XM_010039355.3	Chr11	*Malus domestica*	rna-NM_001294127.1	4
EgWRKY111	rna-XM_010039605.3	Chr11	*Malus domestica*	rna-XM_008389468.4	12
EgWRKY111	rna-XM_010039605.3	Chr11	*Malus domestica*	rna-XM_008371590.4	4
EgWRKY2	rna-XM_039316856.1	Chr01	*Oryza sativa*	rna-XM_015774235.3	3
EgWRKY5	rna-XM_039317100.1	Chr01	*Oryza sativa*	rna-XM_015760956.3	11
EgWRKY5	rna-XM_039317100.1	Chr01	*Oryza sativa*	rna-XM_015764540.3	12
EgWRKY5	rna-XM_039317100.1	Chr01	*Oryza sativa*	rna-XM_026022102.2	1
EgWRKY12	rna-XM_010047269.3	Chr01	*Oryza sativa*	rna-XM_015782638.3	5
EgWRKY12	rna-XM_010047269.3	Chr01	*Oryza sativa*	rna-XM_026024818.2	1
EgWRKY21	rna-XM_010048316.3	Chr02	*Oryza sativa*	rna-XM_015775935.3	3
EgWRKY22	rna-XM_010046160.3	Chr02	*Oryza sativa*	rna-XM_015775479.3	3
EgWRKY22	rna-XM_010046160.3	Chr02	*Oryza sativa*	rna-XM_015791464.3	7
EgWRKY24	rna-XM_010047093.3	Chr02	*Oryza sativa*	rna-NM_001406347.1	1
EgWRKY24	rna-XM_010047093.3	Chr02	*Oryza sativa*	rna-XM_015782350.3	5
EgWRKY25	rna-XM_010049069.3	Chr03	*Oryza sativa*	rna-XM_015779545.3	1
EgWRKY43	rna-XM_010054488.3	Chr05	*Oryza sativa*	rna-XM_015769476.3	4
EgWRKY43	rna-XM_010054488.3	Chr05	*Oryza sativa*	rna-XM_015779547.3	4
EgWRKY55	rna-XM_039315841.1	Chr06	*Oryza sativa*	rna-XM_066303417.1	8
EgWRKY55	rna-XM_039315841.1	Chr06	*Oryza sativa*	rna-XM_015756734.3	9
EgWRKY60	rna-XM_039315439.1	Chr06	*Oryza sativa*	rna-NM_001422763.1	9
EgWRKY60	rna-XM_039315439.1	Chr06	*Oryza sativa*	rna-XM_015786092.3	6
EgWRKY73	rna-XM_010068554.3	Chr07	*Oryza sativa*	rna-XM_015775935.3	3
EgWRKY74	rna-XM_010068568.3	Chr07	*Oryza sativa*	rna-XM_015775479.3	3
EgWRKY74	rna-XM_010068568.3	Chr07	*Oryza sativa*	rna-XM_015791464.3	7
EgWRKY76	rna-XM_010069318.3	Chr07	*Oryza sativa*	rna-XM_015760956.3	11
EgWRKY76	rna-XM_010069318.3	Chr07	*Oryza sativa*	rna-XM_015764540.3	12
EgWRKY77	rna-XM_010069319.3	Chr07	*Oryza sativa*	rna-XM_015774235.3	3
EgWRKY79	rna-XM_010071532.3	Chr08	*Oryza sativa*	rna-XM_015784451.3	5
EgWRKY82	rna-XM_010026581.2	Chr08	*Oryza sativa*	rna-XM_015782638.3	5
EgWRKY94	rna-XM_010031058.3	Chr09	*Oryza sativa*	rna-XM_015791464.3	6
EgWRKY96	rna-XM_039303323.1	Chr10	*Oryza sativa*	rna-XM_015791387.3	7
EgWRKY96	rna-XM_039303323.1	Chr10	*Oryza sativa*	rna-XM_015760950.3	11
EgWRKY96	rna-XM_039303323.1	Chr10	*Oryza sativa*	rna-XM_015764535.3	12
EgWRKY99	rna-XM_039303157.1	Chr10	*Oryza sativa*	rna-XM_015760956.3	11

EgWRKY101	rna-XM_010035731.3	Chr10	*Oryza sativa*	rna-NM_001409358.1	2
EgWRKY107	rna-XM_010037704.3	Chr11	*Oryza sativa*	rna-XM_015774235.3	3
EgWRKY107	rna-XM_010037704.3	Chr11	*Oryza sativa*	rna-XM_015783257.3	5
EgWRKY107	rna-XM_010037704.3	Chr11	*Oryza sativa*	rna-XM_015791387.3	7
EgWRKY107	rna-XM_010037704.3	Chr11	*Oryza sativa*	rna-XM_015760956.3	11
EgWRKY107	rna-XM_010037704.3	Chr11	*Oryza sativa*	rna-XM_015764540.3	12
EgWRKY110	rna-XM_010039355.3	Chr11	*Oryza sativa*	rna-XM_015784892.3	5
EgWRKY12	rna-XM_010047269.3	Chr01	*Zea mays*	rna-XM_008675729.3	3
EgWRKY12	rna-XM_010047269.3	Chr01	*Zea mays*	rna-XM_008657236.4	8
EgWRKY12	rna-XM_010047269.3	Chr01	*Zea mays*	rna-XM_008664711.3	10
EgWRKY15	rna-XM_010055259.3	Chr01	*Zea mays*	rna-NM_001176359.1	3
EgWRKY19	rna-XM_010040631.3	Chr02	*Zea mays*	rna-NM_001153619.2	1
EgWRKY21	rna-XM_010048316.3	Chr02	*Zea mays*	rna-NM_001136605.1	1
EgWRKY22	rna-XM_010046160.3	Chr02	*Zea mays*	rna-XM_008661858.3	9
EgWRKY22	rna-XM_010046160.3	Chr02	*Zea mays*	rna-XM_008658976.4	1
EgWRKY24	rna-XM_010047093.3	Chr02	*Zea mays*	rna-NM_001367370.1	3
EgWRKY43	rna-XM_010054488.3	Chr05	*Zea mays*	rna-NM_001158440.2	2
EgWRKY55	rna-XM_039315841.1	Chr06	*Zea mays*	rna-XM_020552802.3	4
EgWRKY60	rna-XM_039315439.1	Chr06	*Zea mays*	rna-XM_008650811.2	6
EgWRKY60	rna-XM_039315439.1	Chr06	*Zea mays*	rna-NM_001127251.1	7
EgWRKY73	rna-XM_010068554.3	Chr07	*Zea mays*	rna-NM_001136605.1	1
EgWRKY77	rna-XM_010069319.3	Chr07	*Zea mays*	rna-XM_035962691.1	9
EgWRKY94	rna-XM_010031058.3	Chr09	*Zea mays*	rna-XM_008658976.4	1
EgWRKY96	rna-XM_039303323.1	Chr10	*Zea mays*	rna-XM_008655250.4	7
EgWRKY101	rna-XM_010035731.3	Chr10	*Zea mays*	rna-XM_008650811.2	7
EgWRKY107	rna-XM_010037704.3	Chr11	*Zea mays*	rna-NM_001137923.1	4

Table S6. Primers used in the paper.

Constructions	Primer sequences	Amplicon length (bp)
**Primers for qRT-PCR analysis**		
EgUBI3-F	TCACCTACGTCTACCAGAAGG	104
EgUBI3-R	TCCTCGAAAGCTGTAAACATGG	
EgWRKY14-F	AGAGTGAAGAGGGTCGTTAGAG	85
EgWRKY14-R	CTTTGATGGGCTTTTGACCG	
EgWRKY50-F	TGCAGGGTTTCAGATGACG	78
EgWRKY50-R	CTCTTCAGTGAGTACGACAAGG	
EgWRKY77-F	GAGTGAGGTTCGTGAGGTTATG	125
EgWRKY77-R	AGCAACCTCTTCCGTCTTTC	
EgWRKY33-F	AAGTGCAGTACAATGAGAGGG	74
EgWRKY33-R	ATCAGCATCGTTGGATCGTC	
EgWRKY60-F	GAAGCATCTGACACTGGCCT	172
EgWRKY60-R	CCAGAACAGCTTGGTCCTCA	
EgWRKY101-F	GCTGGCTGCTGAAAACAAGA	163
EgWRKY101-R	GCCATTGTCGTTAGTGCTGC	
EgWRKY110-F	GGTTTCTGTGGAGGTGTCGT	188
EgWRKY110-R	CCGGGAGTGTTTCAGAAGGT	
EgWRKY24-F	GGAGCAGAACGGTGAGAGAA	199
EgWRKY24-R	GCCCTTAGATCATGGGATGCT	
EgWRKY52-F	TGGCAAGTTCAGATGGGATG	124
EgWRKY52-R	TCCAATGTGACGGTAGGAAAC	
EgWRKY103-F	CAGGATCAGAAACCCGAGAAC	127
EgWRKY103-R	AGTCTGAGTTATTCGCTGTGG	
EgWRKY100-F	TGAGAATGGGCTTGAAGGAC	125
EgWRKY100-R	AATTCTGGGTGTTACGGTAGG	
EgWRKY39-F	CATCCAAGCCCGTAGTTCAG	131
EgWRKY39-R	TGAGTCCCCATTTGAAGCTC	
